# Zika virus infection leads to mitochondrial failure, oxidative stress and DNA damage in human iPSC-derived astrocytes

**DOI:** 10.1038/s41598-020-57914-x

**Published:** 2020-01-27

**Authors:** Pítia Flores Ledur, Karina Karmirian, Carolina da Silva Gouveia Pedrosa, Leticia Rocha Quintino Souza, Gabriela Assis-de-Lemos, Thiago Martino Martins, Jéssica de Cassia Cavalheiro Gomes Ferreira, Gabriel Ferreira de Azevedo Reis, Eduardo Santos Silva, Débora Silva, José Alexandre Salerno, Isis Moraes Ornelas, Sylvie Devalle, Rodrigo Furtado Madeiro da Costa, Livia Goto-Silva, Luiza Mendonça Higa, Adriana Melo, Amilcar Tanuri, Leila Chimelli, Marcos Massao Murata, Patrícia Pestana Garcez, Eduardo Cremonese Filippi-Chiela, Antonio Galina, Helena Lobo Borges, Stevens Kastrup Rehen

**Affiliations:** 1 D’Or Institute for Research and Education, Rio de Janeiro, Brazil; 2 Institute of Biomedical Sciences, Federal University of Rio de Janeiro (UFRJ), Rio de Janeiro, RJ Brazil; 3 Institute of Medical Biochemistry Leopoldo De Meis, Federal University of Rio de Janeiro, Rio de Janeiro, RJ Brazil; 4 Insitute of Biology, Department of Biophysics and Biometrics, State University of Rio de Janeiro (UERJ), Rio de Janeiro, RJ Brazil; 5 Laboratory of Neuropathology, State Institute of Brain Paulo Niemeyer, Rio de Janeiro, RJ Brazil; 6 Institute of Biology, Federal University of Rio de Janeiro (UFRJ), Rio de Janeiro, RJ Brazil; 7 Institute of Health Sciences, Federal University of Rio Grande do Sul (UFRGS), Porto Alegre, RS Brazil; 8 Research Institute Prof. Joaquim Amorim Neto (IPESQ), Campina Grande, PB Brazil

**Keywords:** Viral host response, Cellular neuroscience, Astrocyte

## Abstract

Zika virus (ZIKV) has been extensively studied since it was linked to congenital malformations, and recent research has revealed that astrocytes are targets of ZIKV. However, the consequences of ZIKV infection, especially to this cell type, remain largely unknown, particularly considering integrative studies aiming to understand the crosstalk among key cellular mechanisms and fates involved in the neurotoxicity of the virus. Here, the consequences of ZIKV infection in iPSC-derived astrocytes are presented. Our results show ROS imbalance, mitochondrial defects and DNA breakage, which have been previously linked to neurological disorders. We have also detected glial reactivity, also present in mice and in *post-mortem* brains from infected neonates from the Northeast of Brazil. Given the role of glia in the developing brain, these findings may help to explain the observed effects in congenital Zika syndrome related to neuronal loss and motor deficit.

## Introduction

Zika virus (ZIKV) was discovered in 1947 in Africa and remained quite neglected until occurrences of Zika infection were reported in Micronesia (2007), French Polynesia (2013) and South America (2013)^1–5^. ZIKV had not been tied to developmental disorders back then. In 2015–16, however, the virus was linked to an outbreak of congenital malformations in Brazil and declared a Public Health Emergency of International Concern^1^. Sequencing analysis defined two main strains, the African and the Asian, the latter which thrived in the Americas and was reported to be linked to congenital malformations and Guillain-Barré Syndrome^6–9^.

ZIKV neurotropism is known since its discovery and first description in Uganda^2^. The virus deadly effects have been shown in neural stem cells, in addition to growth impairment of human neurospheres and brain organoids^10–12^. Almost all studies have described ZIKV infection and consequences in neural progenitor cells (NPCs), the first cell target extensively investigated. ZIKV infection disrupts NPC proliferation and differentiation, crucial processes correlated to congenital malformations^12,13^. On the other hand, astrocytes play important roles during neural development and multiple pathological findings in ZIKV-infected fetuses raised questions of possible glial disturbances^14–16^. Recently, studies have focused on the role of astrocytes during ZIKV infection: Retallack and collaborators observed that ZIKV preferentially infected cells with glial and astrocytic markers in human organotypic cultures^17^. Simonin and colleagues described an increased ZIKV infection rate in a human astrocyte cell line when compared to human iPSC-derived NSCs^18^. More recently, primary human astrocytes were shown to be susceptible to ZIKV, with an infection rate of ~ 60% (MOI = 1)^19^. Human fetal astrocytes were also found to be more infected than human fetal neurons, with a higher viral production showing that they probably work as a viral reservoir^20^. Still, little is known about the specific cellular mechanisms disrupted by ZIKV^19–22^. The study of ZIKV biology can be greatly benefitted from the use of human induced pluripotent stem cells (iPSC), which allows the modelling of human development through differentiation into multiple cell types.

ZIKV belongs to the *Flaviviridae* family. Members of this family replicate in the endoplasmic reticulum (ER), with evidences of ZIKV interaction with this organelle^23–25^. The replication of hepatitis C virus (HCV), also a member of *Flaviviridae* family, leads to Ca^+2^ release from the ER lumen to the cytoplasm, increasing the overall production of reactive oxygen species (ROS). Mitochondria might uptake this Ca^+2^, leading to more ROS production^26^. ROS is another common consequence of flavivirus infections^27^, and its increase has been described as a result of ZIKV infection in yeast^28^ and in brains of infected mice^29^.

Other cellular processes, such as proliferation and mitosis, have been described as affected by ZIKV^11,30,31^. Mitosis defects happen due to deficient karyokinesis or chromosomal breakage, often leading to mitotic catastrophe or apoptosis to avoid chromosomal instability^32^. DNA breakage, in turn, can be caused by ROS generated during the viral replication process and can affect one strand of DNA (single-strand break, SSB) or both (double-strand break, DSB)^33^. The DNA damage response (DDR) activates repair pathways leading to the expression of genes used to monitor DNA damage. Viral structures can also trigger DDR by interacting with host DNA^33^. ZIKV has been shown to trigger DDR in human neural stem cells (NSCs)^34^, and proteomics and RNA-seq analysis demonstrated that ZIKV-infected neurospheres upregulate BRCA1 and MRE11A^35^. BRCA1 has been linked to DSBs^36^, and it interacts with phosphorylated histone H2AX (γH2AX), a classical DSB marker^37^.

Here, cellular consequences of ZIKV infection in iPSC-derived astrocytes are investigated. Astrocytes are one of the cell types with the highest infection rate in the brain. ZIKV triggers mitochondrial damage and increased ROS levels, which culminate in DNA breaks, probably the virus final denouement leading to cell death. Moreover, ROS scavenging helps to protect astrocytes against DNA damage by reducing apoptotic and mitotic catastrophe nuclei features. Our *in vitro* reactive gliosis model showed an increase in GFAP fluorescence intensity, which was also observed *in vivo* and in *post-mortem* tissue analysis. These findings complement previous data published to solve the puzzle related to mechanisms of ZIKV infection and its cellular outcomes.

## Results

### Human iPSC-derived astrocytes are preferentially infected when compared to iPSC-derived neural stem cells and neurons

**Figure 1:**
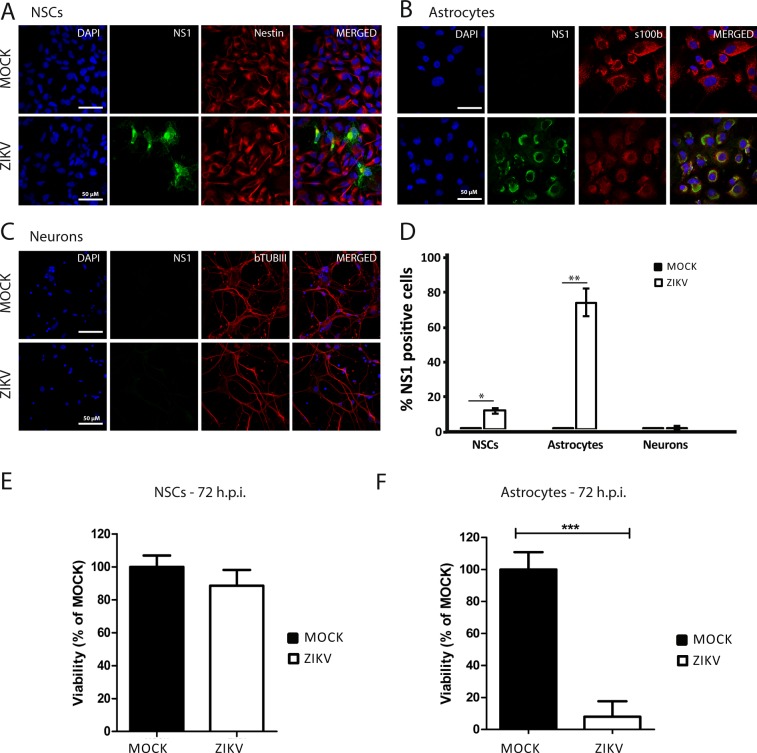
ZIKV infection rate of human brain cells derived from iPSC. (**A**–**C**). Cells were infected at a multiplicity of infection (MOI) = 1 and analyzed 72 hours post infection (hpi). (**A**). Immunofluorescence images of NSCs stained for ZIKV NS1 protein (green) and Nestin (red). (**B**) Astrocytes stained for ZIKV NS1 protein (green) and S100b (red). (**C**) Neurons stained for ZIKV NS1 protein (green) and beta tubulin III (red). Blue channel is DAPI staining for all cell types. Scale bar: 50 µm. (**D**). Quantification of the rate of infection according to cell type (Data is shown as average ± SEM. For NSCs, n = 3, derived from iPS lines GM23279A, CF1 and CF2, *p = 0.026; for astrocytes, n = 4, derived from iPS lines GM23279A, CF1, CF2 and C15, **p = 0.003; for neurons, n = 3, derived from iPS lines GM23279A, CF1 and CF2, non-significant). (**E**) Cell viability assay (MTT) for MOCK and ZIKV-infected NSCs, non-significant, n = 3. (**F**) MTT for MOCK and ZIKV-infected astrocytes, n = 3, ***p < 0.001. Data is shown as average ± SEM.

Zika virus targeting of brain cells varies in the literature according to the viral strain as well as the model employed^18,22^. Here we used human iPSC-derived brain cells to investigate which cell types are most infected by ZIKV. Neural stem cells and neurons were generated as previously described^38,39^. Astrocyte differentiation followed the protocol described by *Yan et al., 2013*^39^ and exhibited the main astrocytic markers (Supplementary Material – Fig. S1). Infection rate was assessed in human iPSC-derived NSCs, astrocytes and neurons with the Asian ZIKV strain PE, isolated from Brazil^40^. Infection efficiencies were evaluated by immunofluorescence of infected cells at 72 hours post infection (hpi) with a multiplicity of infection (MOI) of 1. ZIKV shows tropism for glial cells, infecting around 80% of astrocytes, 20% of NSCs and a minority of neurons (about 2%) (Fig. 1A–D). To further confirm astrocytic tropism, at 72 hpi cell viability of NSCs and astrocytes clearly correlates with infection efficiency rates (*ρ* = −0.99) (Fig. 1E,F). Moreover, a nuclear morphometric analysis (NMA) of infected cells was performed in each cell type. Nuclear morphometry can be used to objectively evaluate cellular mechanisms such as proliferation, mitotic catastrophe and apoptosis^41^. Here, NMA shows that ZIKV-infected astrocytes have an increased number of cells with small regular (SR) and irregular (I) nuclei, nuclear phenotypes that are typical of apoptosis and mitotic defects, respectively. NSCs showed less nuclei alterations when compared to astrocytes while no alterations were found in neurons nuclei, which strongly correlates to less infection (Fig. S2A). We have also checked infection rate *per* cell type in a mouse model of ZIKV infection. Cortical astrocytes (ALDH1L1 + /NS1 +  = 13.3%, SE = 1.03) are more infected when compared to neurons (NeuN + /NS1 +  = 4.6%, SE = 0.7) and oligodendrocyte lineage cells (Olig2 + /NS1 +  = 6.5%, SE = 0.49) in the cingulate cortex (Fig. S3). NSCs were evaluated in the hippocampus and 7.6% (SE = 2.3) of SOX2 positive cells were found infected (Fig. S3). Not surprisingly, we found that microglial cells, the first immune sentinels of the CNS, are considerably infected *in vivo* (IBA1 + /NS1 +  = 34.6%, SE = 3.52) (Fig. S3). Together with microglia, astrocytes represent the most infected cell types in mice. Besides, among the human iPSC-derived cell types tested, astrocytes are also a preferential target for ZIKV. Therefore, we have decided to further investigate the consequences of ZIKV infection in astrocytes considering its important role on cortex development and neural progenitor migration.

### ZIKV impairs mitochondrial metabolism in infected human astrocytes

**Figure 2:**
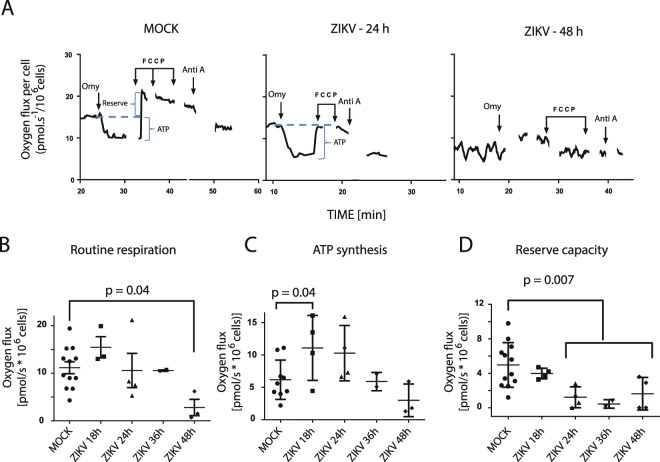
ZIKV-induced mitochondrial dysfunction. (**A**) Respirometry analysis of MOCK or ZIKV-infected astrocytes after 24 and 48 hpi. (**B**) Routine respiration, (**C**) ATP synthesis, (**D**) Reserve capacity of infected astrocytes at different time points post infection. N = 12 for MOCK and N = 3 for each time point of ZIKV infection. Astrocyte lines used were derived from iPSC lines GM23279A, C15, CF1 and CF2.

Upon cell entry, flaviviruses release their genetic material into the cytoplasm, which associates to ER membranes and starts synthesizing viral proteins inside ER invaginations^27,42^. An ER function alteration might indicate an early stage of cell death preceding calcium imbalance, mitochondrial dysfunction and accumulation of ROS^43,44^. Furthermore, energy demanded by the replication process may burden mitochondria, causing stress to these organelles. To analyze the impact of such alterations in cell metabolism and ATP synthesis, a respirometry analysis in ZIKV-infected astrocytes was performed (72 hpi and earlier) to analyze early and late events before cell death (Fig. 2). Within 18 to 24 hpi, there was a transient two-fold increase in oxygen flux coupled to ATP synthesis, which may correspond to the initial viral effect of improving energy production to its own replication. At 36 hpi, the rate of oxygen flux coupled to ATP synthesis returned to the levels of MOCK. At approximately 48 hpi, there was a tendency of decrease in oxygen flux coupled to ATP synthesis (Fig. 2C). Despite the initial effect observed in ATP synthesis after infection (18 and 24 hpi), the reserve capacity was significantly lower 24 hours after ZIKV infection (Fig. 2D), which suggests an energy stress of mitochondria. In fact, after 48 hours ZIKV caused a severe impairment in mitochondrial function, as observed by the reduction of routine respiration (Fig. 2B). Even in more resistant cells, a transient stimulation of oxygen flux coupled to ATP synthesis was observed 24 hours after infection and, at longer times, mitochondrial function was consistently impaired. We have further observed mitochondrial alterations in electron microscopy of infected cells, such as disrupted cristae and outer membranes, with darker electron density (Fig. S4).

### ZIKV infection leads to the production of ROS in infected human astrocytes

**Figure 3:**
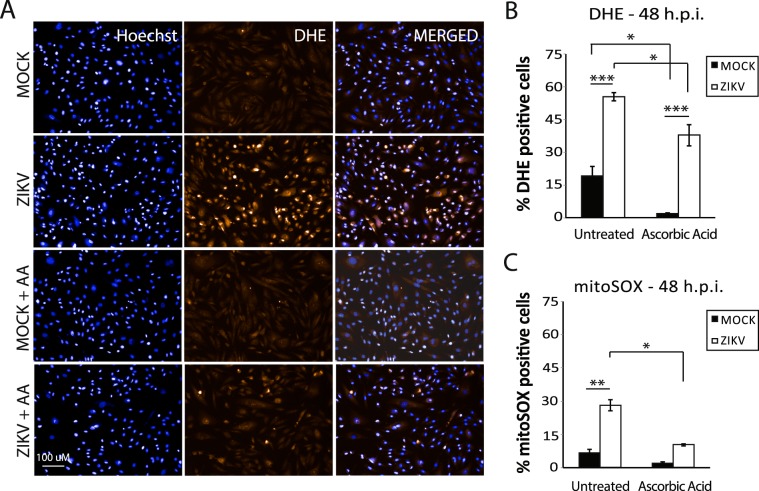
ROS production in ZIKV infection. (**A**) Staining of ROS superoxide indicator dye DHE in MOCK and ZIKV-infected astrocytes (untreated and treated with ascorbic acid 80 µM). Hoechst was used for nuclei staining. N = 3. Scale bar: 100 µm. (**B**) Quantification of DHE staining (shown as the percentage of DHE-positive cells) in MOCK and ZIKV-infected astrocytes treated or untreated with ascorbic acid. N = 3. (**C**) Quantification of mitoSOX ROS superoxide indicator dye staining (shown as the percentage of mitoSOX-positive cells) in MOCK and ZIKV-infected astrocytes untreated or treated with ascorbic acid. N = 3. Data is shown as average ± SEM; *p < 0.05, **p < 0.01, ***p < 0.001. Astrocytes derived from iPSC lines GM23279A, CF1 and C15 were used.

Calcium (Ca^2+^) released from the ER lumen to cytoplasm can be uptaken by mitochondria, leading to an increase in the production of ROS^26^. Moreover, mitochondria are believed to be the main site for ROS production inside the cell^43^. Regarding mitochondrial impairment observed at 48hpi, total and mitochondrial ROS production were analyzed through the superoxide indicator dihydroethidium (DHE) and mitoSOX^TM^, respectively, at the same timepoint. Astrocytes show 55% DHE staining when infected by ZIKV, a 2.9-fold increase when compared to MOCK (Fig. 3A,B), while MitoSOX^TM^ staining increased by 4.2-fold in infected cells (Fig. 3C). Ascorbic acid, a known ROS scavenger, reduced DHE and mitoSOX staining in astrocytes (Fig. 3A–C and Supplementary Fig. S5). Interestingly, NMA suggested that ROS may be important for the cellular toxicity of ZIKV. Considering infected cells with high DHE staining (>Average + 1 SD), around 60% exhibited irregular nuclei (the nuclear phenotype that indicates mitotic catastrophe) and 96% of small regular nuclei (the nuclear phenotype that indicates apoptosis) (Fig. S2B,C).

### ZIKV causes DNA breaks and activates DDR signaling in infected human astrocytes

**Figure 4:**
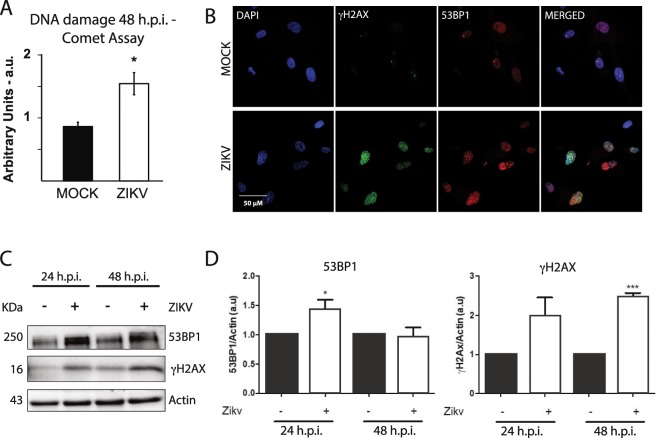
ZIKV-induced DNA damage. (**A**) Alkaline comet assay shows an increase in DNA damage in ZIKV-infected astrocytes when compared to MOCK. N = 4. (**B**) γH2AX and 53BP1 immunostaining in MOCK- and ZIKV-infected astrocytes, 48 h.p.i.. DAPI for nuclei staining is shown in blue, γH2AX is shown in green, and 53BP1 is shown in red. Merged images are shown at the right. Scale bar: 50 µm. Quantification of images is shown on Fig. 5C. (**C**) Western blotting (WB) analysis of γH2AX and 53BP1 in MOCK and ZIKV-infected astrocytes at 24 and 48 hpi, MOI = 1. (**D**) Protein levels were quantified related to actin expression levels. N = 3. *p < 0.05, ***p < 0.001.

**Figure 5:**
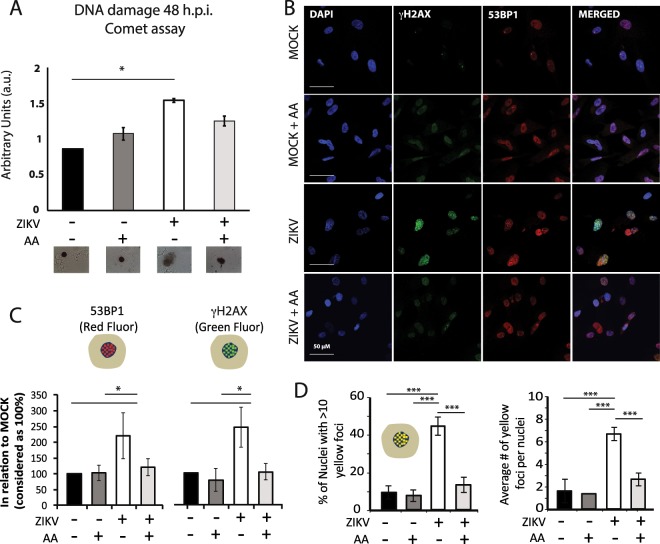
Ascorbic acid attenuates DNA damage. (**A**) Alkaline comet assay. N = 4. *p = 0.02. (**B**) Ascorbic acid treatment in ZIKV-infected astrocytes, stained for DDR proteins γH2AX and 53BP1. (**C**) Quantification of γH2AX and 53BP1 fluorescence levels in MOCK- and ZIKV-infected astrocytes treated and untreated with ascorbic acid. Over 200 nuclei were quantified per condition. Scale bar: 50 µM. (**D**) Number co-localized foci of γH2AX and 53BP1 were quantified in the same astrocyte nuclei analyzed in C. *p < 0.05, ***p < 0.001. Astrocytes derived from iPSC lines GM23279A, CF1 and C15 were used.

ROS are important causes of DNA breaks, being the second major cause of DSBs following ionizing radiation^45,46^. Since ROS production was observed in ZIKV-infected cells, we next evaluated DNA damage. First, through comet assay, an increase in total DNA breaks was found in ZIKV-infected astrocytes when compared to MOCK at 48 hpi (Fig. 4A). We then wondered whether ZIKV-induced DNA breaks triggered a DDR in infected cells. It has been previously shown that astrocytes downregulate DDR genes, even though they are still capable of phosphorylating H2AX upon DNA damage^47^. The expression of key components of the DDR cascade in astrocytes was then evaluated. Histone H2AX phosphorylation after DNA damage (named γH2AX) is an event that precedes the recruitment of other DNA repair proteins, such as 53BP1, which interacts with many DSB-responsive proteins^48,49^. γH2AX and 53BP1 protein levels are increased after 24 and 48 hpi, as quantified by Western Blotting (Figs. 4C,D and S7). Moreover, the two proteins are observed as nuclear foci in ZIKV-infected astrocytes, as seen by immunofluorescence analysis, and they co-localize (Figs. 4B and 5C,D), an indication that they are acting together at a DNA break site.

### ZIKV-induced DNA damage can be rescued by the ROS scavenger ascorbic acid

Ascorbic acid reduced ROS levels in infected astrocytes (Fig. 3). As ROS cause DNA breaks, DNA damage in ZIKV infection was estimated when cells were treated daily with this antioxidant until 48 hpi. Ascorbic acid reduced total DNA breaks as measured by comet assay (Fig. 5A). Ascorbic acid also decreased the intensity of 53BP1 and γH2AX expression induced by ZIKV (Fig. 5B,C), as well as the percentage of nuclei with more than 10 co-localized dots and the average number of co-localized foci (Fig. 5D). Finally, ascorbic acid also reduced the percentage of irregular and small regular nuclei induced by ZIKV, suggesting that oxidative stress induced by ZIKV may be involved in the mitotic catastrophe and apoptosis induced by the virus (Fig. S2D). It is interesting to note that the reduction of ROS levels as seen by DHE and mitoSOX superoxide indicators in ascorbic acid treatment (Fig. 3) was not as sharp as nuclei morphology improvements and 53BP1/γH2AX foci number rescue in treated cells. It was previously suggested that a damage threshold could define whether DNA breaks are repaired or not. Low damage levels could allow repair and cell survival, while high damage levels could drive cell fate towards apoptosis^50^. Thus, it is plausible that the decrease of ROS by ascorbic acid was enough to reduce ZIKV-induced DNA damage, but even though ascorbic acid incubation significantly reduced ROS levels and DNA breaks post infection, it was not enough to avoid cell death. At 48 hpi, cell viability was not increased by ascorbic acid and no effect was seen in infection rate (data not shown).

### ZIKV infection induces glial reactive state

**Figure 6:**
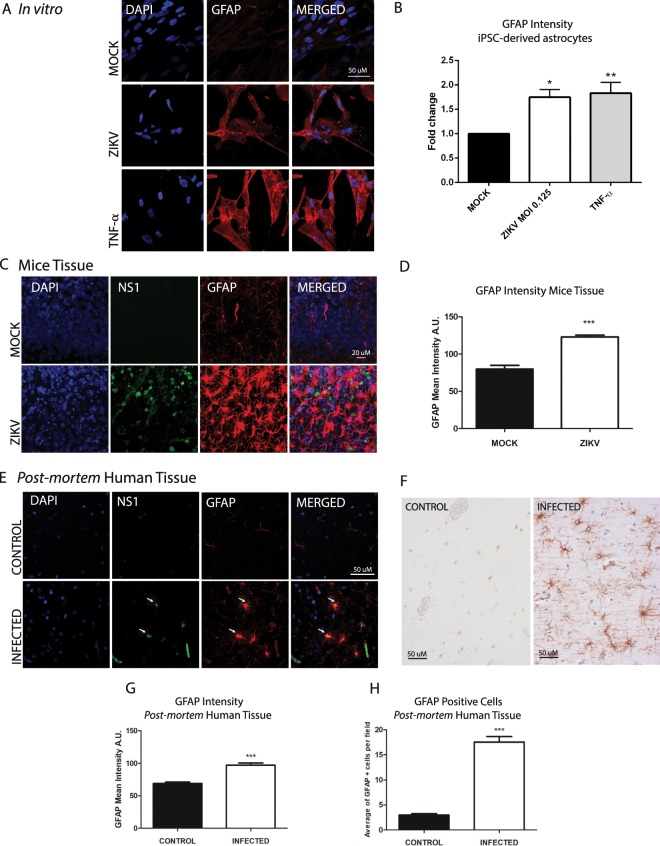
Astrogliosis induced by ZIKV infection. (**A**) Immunofluorescence images of MOCK-, ZIKV-infected iPSC-derived astrocytes and TNF-α condition as positive control. DAPI (blue) and GFAP (red). Scale bar: 50 μm. (**B**) Quantification of GFAP intensity in MOCK- and ZIKV-infected astrocytes at 4 dpi, MOI = 0.125. Quantification of TNF-α (10 ng/ml) condition as positive control of gliosis assay. N = 4. *p = 0.015, **p = 0.0085. (**C**) Immunofluorescence images of ZIKV-infected mice brains. DAPI (blue), ZIKV NS1 protein (green) and GFAP (red). Scale bar: 20 µm. (**D**) Quantification of GFAP intensity in MOCK- and ZIKV-infected 7 dpi mice cingulate cortex. N = 3. (**E**) Immunofluorescence images of control and infected *post-mortem* human brains. DAPI (blue), ZIKV NS1 protein (green) and GFAP (red). White arrows indicate GFAP positive infected cells. Scale bar: 50 µm. (**F**) Immunohistochemistry for GFAP in control and infected *post-mortem* human brains. (**G,H**) Quantification of GFAP intensity and average number of GFAP positive cells in *post-mortem* human tissue, respectively. Data is shown as average ± SEM; ***p < 0.0001.

Reactive gliosis is a common and complex consequence induced by multiple stressor agents and neuroinflammatory conditions, such as bacterial and viral infections, neurodegenerative diseases and acute trauma^51^. Viral antigens can lead to an inflammatory environment that, through microglia activation, culminate to astrocytic reactivity^21^. During the active state, astrocyte morphology and cytoskeleton protein expression are altered, showing hypertrophy, higher number of cytoplasmic processes and increased expression of intermediate filaments, especially GFAP, usually detected by increased marker intensity^52^. To evaluate GFAP expression we infected iPSC-derived astrocytes for 4 days with reduced MOI = 0.125 to minimize cell death (Fig. 6A,B). We observed a significant increase of 1.74-fold in GFAP intensity in infected cells, similar to the one induced by TNF-α (1.83-fold increase), a proinflammatory cytokine routinely used as a positive control for astrocyte activation^53^. Vimentin intensity was also 2.59-fold increased after ZIKV infection. (Fig. S6). GFAP and vimentin increase found in infected astrocytes suggest that ZIKV infection could be actively inducing a reactive state (Fig. S6). C57BL/6 mice infected with ZIKV (same viral strain used to *in vitro* infections) at P0 exhibited 54% increase of GFAP staining intensity at P7 comparing to MOCK mice, an indication that these cells are activated in a process likely triggered by ZIKV (Fig. 6C,D). This reactivity pattern was confirmed in the frontal lobe of neonates with congenital Zika syndrome (CZS) from the state of Paraíba (Brazil) who died shortly after birth (Fig. 6E,F). Infected brain tissue showed 40.4% increase in GFAP intensity when compared to an age-matched non-infected neonate brain, indicating the presence of reactive astrocytes in a similar way as observed in mice brain tissue (Fig. 6G). GFAP positive cells were found to be 5.8-fold increased in ZIKV-infected postmortem tissue, another classical feature of reactive gliosis^51^ (Fig. 6H). Therefore, ZIKV infection of astrocytes not only leads to multiple severe consequences, but it also induces reactive gliosis. Considering that a reactive state can be induced in astrocytes by viral infection itself or by released viral proteins, cytokines and immune triggers likely present in neonate brains with CZS, it is plausible to suggest that astrogliosis could be induced by both mechanisms^52^.

## Discussion

The first studies about ZIKV primarily focused on neural progenitor and neural stem cells, though more recently a predilection of the virus for glial cells has been described^17,18,29^. Besides these recent findings, little is known regarding the consequences of infection to astrocytes. Here, a preference of ZIKV for astrocytes was confirmed when compared to NSCs and neurons in iPSC-derived cells. We also evaluated infection rates *in vivo*. The analysis of cingulate cortex of infected mice suggests that astrocytes are preferentially infected when compared to NSCs, neurons and oligodendrocytes, although microglia seems to have a higher infection rate (Supplementary Fig. S3). A pronounced infection in microglia is not surprising, given the role of this cell type in immune response. Meertens *et al*., 2017 have shown that both microglial cells and astrocytes express Axl receptor^54^, which has been shown to mediate ZIKV cell entry^54–56^. Though we have not evaluated Axl expression here, our data matches these findings. Interestingly, ZIKV infected microglia was shown to induce astrogenesis at the expense of neurogenesis^57^, therefore favoring its target cells. In addition to this point, we have also found a 2.3-fold increase in the percentage of IBA1 positive cells comparing MOCK- and ZIKV-infected mice, clearly indicating classical microglial activation following infection (data not shown).

Regarding ZIKV effect in astrocytes, we have shown that ZIKV induced a pronounced glial reactivity in all models tested (*in vitro* and *in vivo*, both in mice and in human brain tissue), corroborating previous findings^16,58^. It is likely correlated to an increased expression of pro-inflammatory chemokines and cytokines already described in infected astrocytes^19^. These may explain malformations observed in infected neonates, such as dysplastic cortex and over migration to subarachnoid spaces in brains^14^, probably due to the damage of radial glia and limitant glia, respectively. In fact, proteomic analysis of ZIKV-infected human astrocytoma cell line (U-251) at 48 hpi showed disruption in pathways involved in cell migration and proliferation, and in the ones related to synaptic plasticity^59^. Considering that astrocytes play crucial roles during brain development as well as in post-natal and adult stages, such as blood-brain barrier (BBB) maintenance, metabolic neuronal support, guidance for neural progenitor migration, and synaptic regulation, their dysregulation could have severe implications not only for congenital diseases, such as microcephaly, but also for the onset of neurologic disorders such as Parkinson’s disease (PD) and Alzheimer’s disease (AD)^60–62^. Further investigations are needed to determine whether surviving children infected by ZIKV might show an increased rate of neurological disorders later in life. It is important to mention that astrogliosis triggered by viral infection remains longer when compared to gliosis induced by acute trauma^52^, which increases the relevance of studying possible long-term consequences of viral infection.

Several flaviviruses cause ER stress and ROS increase as consequences of viral replication^63–65^. A recent study showed that ZIKV promotes ER stress and UPR, though the authors did not explore an associated increase in intracellular ROS^25^. ER membranes are used by flaviviruses to form vesicle packets (VPs) or double membrane vesicles for viral genome replication. ER disruption can induce Ca^+2^ influx that has been linked to virus-induced rearrangement of the ER membrane^42^. Areas of close contact between ER and mitochondria, called mitochondria associated membranes (MAM), enable the exchange of molecular chaperones and Ca^+2^ between the two organelles, a process that promotes the synthesis of ROS and is critical for mitochondrial-dependent induction of cell death^43,44,66^. ROS increase may also result from mitochondrial overload, as ZIKV energy requirements for replication increase ATP synthesis by OxPhos, boosting oxygen flux coupled to ATP synthesis at 18 hpi with no apparent impairment of reserve capacity. This increase may be critical in tissues with high energy demand, such as the central nervous system (CNS), which is efficient for a basal ratio of ATP synthesis and mitochondrial reserve capacity, adjusted for situations of rapid rise in ATP. At 24 hpi, the increased necessity for ATP persists, but a decrease in reserve capacity indicates the first signs of stress, culminating in mitochondrial failure at 48 hpi as observed by the reduction in routine respiration and by mitochondrial damage seen in electron microscopy. Conditions where mitochondrial reserve capacity does not support ATP requirements are associated with cell death in multiple tissues^67^. Moreover, mitochondrial dysfunction in astrocytes causes apoptosis in motor neurons, and ROS-induced mitochondrial damage is responsible for axon destruction in Guillain-Barré Syndrome, a known complication of ZIKV infection, as well as in multiple sclerosis, causing demyelination^68^.

Oxidative stress has been implicated in increased risk for neural tube defects. Micronutrients with antioxidant capacities such as vitamins C and E may help reduce the risk of birth defects^69^. Therefore, antioxidant supplements and an increased dietary intake of antioxidant-rich foods could potentially benefit ZIKV-infected pregnant women. ROS-induced DNA damage is involved in DDR-defective disorders, which are characterized in many cases by impaired development, including microcephaly^70^. Among DNA breaks, DSBs are the most cytotoxic ones and can be repaired by two distinct paths: homologous recombination (HR) and non-homologous end joining (NHEJ). NHEJ is more common as it can be activated at any phase of the cell cycle, while HR is active during S-G2 phases^45,71^. Astrocytes are differentiated, less proliferative cells and tend to activate NHEJ to repair their genetic material. NHEJ does not require the pairing of a homologous chromosome^47^, making it less accurate than HR; therefore, it could trigger chromosomal instability and numeric and structural issues, if defectively repaired cells survive^72,73^. Unrepaired DSBs can lead to microcephaly through loss of proliferative potential, as they could cause checkpoint arrest or the inability of cells to replicate efficiently^70,74,75^. Autosomal recessive primary microcephaly (MCPH) is caused by reduced neural proliferation during embryonic development, and the major genetic causes for this condition are mutations in genes that act at the centrosome. 53BP1 responds to centrosome loss as part of a mitotic surveillance pathway (USP28-53BP1-p53-p21 signaling axis), causing cell cycle arrest or cell death in response^74^. Therefore, an increased expression of 53BP1 as seen here could be a reflex not only of DDR mechanisms but also of the mitotic surveillance pathway. The nuclear irregularities described can originate from DNA damage^76^ and potentially result in multipolar spindles, increasing the frequencies of viable aneuploid and polyploid cells^30^, leading to chronic pathologies. In fact, ZIKV-infected human NPCs show mitotic dysfunctions and increased aneuploidy in chromosomes 12 and 17^30^. Interestingly, chromosome 17 has important genes for DDR, such as BRCA1 and TP53^77^. Ascorbic acid reduced DDR markers as well as the percentage of nuclei with apoptotic and mitotic catastrophe features in a very important way, which suggests that ROS underlie these effects.

Finally, ZIKV crosses the BBB^78^, so questions remain as far as consequences to the adult brain, rich in astrocytes. Moreover, could infants born healthy from ZIKV-infected mothers have future complications?^79^ ZIKV infection has been shown to have long-term neuropathological and behavioral consequences in mice^29^. Astrocytes control redox homeostasis in the adult CNS, and their depletion can lead to motor deficits and neuronal loss caused by oxidative stress^80^. PD, AD and other neurodegenerative disorders are characterized by the activation of oxidative stress, glial reactivity and mitochondrial impairment, which have been connected to the pathogenesis of these and other diseases^81,82^, making those questions even more relevant.

## Methods

### Cell culture

Four different lines of human induced pluripotent stem cells were cultured as described^11,39^. GM23279A iPSC line was obtained commercially from Coriell Institute Biobank; CF2 and CF1 lines were generated from fibroblasts, and C15 were generated from urine cells. GM23279A, CF1 and CF2 cell lines were previously published in Casas *et al*.^38^. C15 cell line was published in Trindade *et al*.^53^. Cells were reprogrammed using the CytoTune 2.0 Sendai Reprogramming kit (Thermo Fisher Scientific, USA) as described elsewhere^83,84^. Characterization of the reprogrammed cell lines was conducted by immunostaining of both iPSCs colonies and iPSCs- derived embryoid bodies for self-renewal and three germ layer markers as described previously^83,84^, and shown in Fig. S8. The reprogramming of human cells was approved by the ethics committee of Copa D’Or Hospital (CAAE number 60944916.5.0000.5249, approval number 1.791.182). All human cell experiments were performed in accordance to Copa D’Or Hospital regulation.

iPS cells were used to obtain neural stem cells (NSCs), astrocytes and neurons used in this study. The differentiation of iPS cells into NSCs followed a previously described protocol^39^. iPS cells (2.5 × 10^5^) were plated on 6-well Geltrex-coated plates for the differentiation protocol. During 7 days, neural induction medium (Neurobasal plus 1X Neural Induction Supplement - NIS) were changed every 2 days. After that, NSCs were cultured on Geltrex-coated plates using neural expansion medium (Neurobasal, Advanced DMEM/F12, 2X NIS) until passage 7 to maturation. NSCs were used to experiments from passage 8 to 20 using the following cell densities: 1 × 10^4^ cells/well for 96-well plates; 4 × 10^4^ cells/well for 24-well plates and 5 × 10^5^-1 × 10^6^ cells for 60 mm plates.

NSCs were used to produce neurospheres for the characterization of the potential to differentiate into ectoderm (Fig. S8C, CF1 and CF2 lines). Cells were plated at a density of 3 × 10^6^ cells/well for 6-well plate and maintained for 10 days under rotation at 90 rpm. During this period, neurospheres were cultured under differentiation medium (half DMEM/F12 and half Neurobasal, supplemented with 1X N2 and 1X B27) and media was replaced every 4 days. At day 10, neurospheres were plated on poly-L-ornitin (Sigma-Aldrich, #P3655 10 µg/ml)/Laminin (Life Technologies, #23017-015 2.5 µg/ml) coated coverslips and fixed after 48 hours.

NSCs were differentiated in mixed neuronal culture as follows: NSCs were dissociated using Accutase (MPBio, #AS00004) and plated on poly-L-ornitin (Sigma-Aldrich, #P3655 10 µg/ml)/Laminin (Life Technologies, #23017-015 2.5 µg/ml) coated dishes at high confluency (25,000–30,000 cells/cm2) in NSC media (Neurobasal, Advanced DMEM/F12, 2X NIS) (day 0). On day 2, the media was changed to 1:1 NSC media: neuronal differentiation media. Neuronal differentiation media was composed by Neurobasal medium supplemented with 2% B27, 2 mM Glutamax (Life Technologies, #35050-061), 100 U/mL Penicillin-Streptomycin (Life Technologies, #15140163). At day 4, media was completely changed to neuronal differentiation media and subsequently half of the media was weekly changed until the end of the protocol. Typically, at day 15, cultures were detached with Accutase and replated in poly-L-lysine (1 mg/ml) coated coverslips (9 × 10^4^ cell density) in neuronal differentiation media containing 10 µM ROCK inhibitor (MERCK Millipore, #688000). On the following day, media was changed to remove iROCK. Neurons were cultured until day 30–35 to perform experiments and viral infection.

Astrocyte culture were also derived from NSCs as described by Yan *et al*.^39^ and recently functional features were detailed by Trindade *et al*.^53^. NSCs were plated on Geltrex-coated 25 cm² culture flask at density of 1.25 × 10^6^ in NSC expansion medium. On the following day, medium was replaced to astrocyte induction medium [AIM - DMEM/F12 (11330-032 – Thermo Fisher Scientific, MA, USA), N2 supplement (17502001 – Thermo Fisher Scientific, MA, USA) and 1% fetal bovine serum - FBS (12657029 – Thermo Fisher Scientific, MA, USA)] and changed every other day during 21 days. Cells were replated in fresh Geltrex-coated flasks at a dilution of 1:4 when confluence was reached. At the end of this period, medium was replaced to astrocyte medium (DMEM/F12 10% FBS) and no coating was added prior to passaging. Astrocytes were cultured for at least 5 weeks to obtain mature cells and perform experiments. Cells were plated using the following cell densities: 2,5 × 10³ cells/well on 96-well plates and 15 × 10³ cells/well on 24-well plates.

### Brain organoid formation

Formation of brain organoids was performed as previously described (Garcez *et al*., 2016)^11^. Briefly, human iPS cells were dissociated, inoculated into a spinner flask at 40 rpm with 50 mL of mTeSR1 supplemented with 10 μM Y-27632 Rho-associated protein kinases inhibitor (iRock - Merck-Millipore, Germany, # 688000). 24 hours later, medium was changed to DMEM/F12 (Thermo Fisher Scientific, #11330057), supplemented with 20% KnockOut^TM^ Serum Replacement (KoSR, Life Technologies, #10828-028), 2 mM Glutamax (Life Technologies, #35050-061), 1% MEM non-essential amino acids (MEM-NEAA, Life Technologies, #11140-050), 55 μM 2-Mercaptoethanol (Life Technologies, #21985023) and 100 U/mL Penicillin-Streptomycin (Life Technologies, #15140163). On day 6, embryoid bodies had the media changed to neural induction media (DMEM/F12, 1X N2 supplement, 2 mM Glutamax, 1% MEM-NEAA and 1 μg/mL heparin (Sigma- Aldrich Corporation, #H3149-100KU) for 5 days. On day 11, cell aggregates were embedded in Matrigel (Corning, #356234) for 1 h at 37 °C and 5% CO2. After, medium was changed to 1:1 DMEM/F12: Neurobasal (Life Technologies, #21103-049), 0.5X N2, 1X B27 minus vitamin A (Life Technologies, #12587-010), 2 mM Glutamax, 0.5% MEM-NEAA, 0.2 μM 2- Mercaptoethanol and 2.5 μg/mL insulin. Four days later, cell aggregates were grown in neuronal differentiation media, composed as aforementioned except by replacing with B27 containing vitamin A (Thermo Fisher Scientific, USA). Medium was changed every week. Organoids were grown until the desired time points and then infected with ZIKV for 72 hours.

### ZIKV production and infection

ZIKV was isolated from a Brazilian sample from the state of Pernambuco (PE)^40^, and was propagated in Aedes albopictus C6/36 cell line. Cells were cultured in Leibovitz’s L-15 medium (Thermo Fisher Scientific, USA) supplemented with 0.3% tryptose phosphate broth (Sigma-Aldrich, USA), 2 mM L-glutamine (Life Technologies, # 21051-024) and 1X MEM non-essential amino acid and 5% FBS. C6/36 cells were infected at a MOI of 0.01 and cultured with L15 supplemented with 2% FBS. Conditioned medium was collected at 6 days post infection, centrifuged at 300 x g, filtered to remove cellular debris and stored at −80 °C.

Virus was tittered in Vero cells by plaque assay. Cells were seeded in 12-well plates and inoculated with 200 μL of 10-fold serial dilutions of viral stocks or samples and incubated at 37 °C. After 1 hour, inoculum was removed and semisolid medium (alpha-MEM containing 1.25% carboxymethylcellulose supplemented with 1% FBS) was added to each well and incubated for 5 days. Cells were fixed with 4% formaldehyde and stained with 1% crystal violet in 20% ethanol solution. Virus titers were expressed as plaque-forming units (PFU) per milliliter and stored in aliquots in −80 °C until use.

Before infection, target cells were washed twice with 1X PBS and incubated with the viral supernatant in the desired MOI for 2 hours. MOCK-infected cells were incubated with conditioned media from uninfected cells prepared exactly as performed for viral propagation for the same amount of time. After that, virus and MOCK supernatants were removed and cells were incubated with the respective media.

### *In vivo* assay

All *in vivo* experiments were performed in accordance and previously approved by the institutional ethical committee at the Federal University of Rio de Janeiro (UFRJ) under protocol number 040-2019. C57BL/6 mice were infected with 10^4^ PFU of ZIKV through intraventricular injection at P0. For MOCK-infected animals, the corresponding volume of MOCK conditioned media (from Vero cells) was injected. At P7 mice were perfused with 4% PFA and the cingulate cortex was analyzed through immunofluorescence. At least three cortical sections were analyzed by immunofluorescence, from three different litters.

### Human tissue analysis

This study was approved by the local internal review board (IRB) under protocol number 52888616.4.0000.5693 for ZIKV affected neonates who died shortly after birth. Control tissue was obtained from the Maternity School from UFRJ, the project was approved and registered under protocol number 1705093. All mothers gave their consent to the autopsies and tissue collection of neonates. Pregnant women were followed at the out-patients’ clinic specialized in arboviroses at IPESQ (*Professor Joaquim Amorim Neto* Research Institute, *Campina Grande* city, *Paraíba* state, Brazil). Control brains were obtained from the Maternity School from UFRJ (protocol number 1705093). Informed consent was obtained from all parents of participants included in the study. All human tissue experiments were performed in accordance to the regulations of the institutions above. Samples from three infected neonates (two at term and one born at 36 gestational weeks) were used. Details regarding neuropathological findings for each case have been previously described^14^. Immuno-histochemical reactions were performed in paraffin embedded tissue from selected areas of the cerebral hemispheres using anti-glial fibrillary acidic protein (GFAP) monoclonal antibody (Cell Marque), clone EP672y (1:500). Tissue Section 5 µm thick were processed for antigen retrieval, peroxidase blocking and then incubated with primary antibody overnight at 4 °C, rinsed in PBS and incubated with Polymer Hi Def (horseradish peroxidase system) for 10 minutes at room temperature (RT), preceded by several washes in PBS. Peroxidase reaction was visualized with DAB substrate, rinsed in running water, and sections were counterstained with Meyer’s hematoxylin and mounted in resinous medium. Immunofluorescence staining for GFAP (clone EP672y; 1:300) and NS1 was performed after antigen retrieval following a standard protocol described in *Immunostaining* section.

### Ascorbic acid treatment

Astrocytes were incubated with viral supernatant or MOCK supernatant for 2 hours as described (MOI 1). Cells were then washed once with PBS 1X, and fresh media was added. Media was supplemented with 80 µM ascorbic acid daily for 48–72 hours.

### Immunostaining

Cells plated in treated glass coverslips were washed twice with PBS 1X, fixed with 4% paraformaldehyde (PFA) for 20 minutes and blocked for 1 hour in blocking buffer - BB (PBS 1X with 5% goat serum – NGS- from Sigma-Aldrich Corporation, #G9023, or PBS 1X plus 0.1% triton with 5% NGS when permeabilization was needed). Primary antibodies were diluted in BB and incubated in coverslips overnight. Coverslips were washed 3 times with PBS 1X, and incubated with secondary antibodies diluted in PBS for 2 hours at RT. Coverslips were washed once and incubated with 300 nM DAPI for 5 minutes, washed again and mounted on glass slides. Quantification of monolayer cells was performed manually on Image J software.

### Western blot

For western blotting, cells were washed with cold PBS and protein was isolated by extraction in UTB Buffer (9 M urea; 75 mM Tris-HCl pH 7.5). The extracted protein (40 µg of protein per sample) was diluted in sample buffer (10% SDS; 10 mM ß-mercaptoethanol; 20% glycerol; 0.2 M Tris-HCl, pH 6.8; and 0.05% bromophenol blue) and electrophoresed in SDS-PAGE for 2 h at 80 V. Gels were then equilibrated in cold transfer buffer (25 mM Tris, 192 mM glycine, 20% methanol) for 15 min. Nitrocellulose membrane was used; transfer was performed overnight at 30 V. For GFAP detection, cells were washed with cold PBS, dissociated with Tryple, pelleted and frozen. Cells were lysed with sample buffer without bromophenol blue, proteins quantified, and bromophenol blue was then added just before electrophoresis. Electrophoresis was conducted as described above and proteins transferred to a PVDF membrane for 1 hour. Antibodies used were monoclonal anti-γ-H2AX phosphorylated at S139 (1:10000, Millipore # 05-636), polyclonal anti-53BP1 (1:1000, Bethyl laboratories # A300-272a), monoclonal anti-GFAP (1:500; Neuromics MO15052) and anti-actin (1:1000, Millipore # MAB1501). Protein bands were visualized using the Luminata Western HRP substrate reagent (Millipore, Brasil) and Chemidoc system (Bio-Rad, Brasil). The band densities related to Actin was quantified using Image J software (NIH, Bethesda, MD, USA). Full-length membranes are displayed in Supplementary Fig. 7.

### Respirometry

Mitochondrial function was assessed by high-resolution respirometry using an Oroboros O2k Oxygraph at 37 °C. DatLab software (Oroboros Instruments, Innsbruck, Austria) was used for data acquisition and analysis. Astrocytes were enzymatically detached from the plate, diluted in culture medium, and seeded to the Oroboros at an approximate concentration of 1 × 10^6^ cells/mL. The routine respiration of cells, measured before the addition of modulators of mitochondrial function, was determined after stabilization of the steady state of oxygen consumption for 10–15 min. Subsequently, ATP synthesis was inhibited with 0.1 µg/mL oligomycin. Oxygen flux coupled to ATP synthesis was determined by the difference between routine respiration and oligomycin-insensitive respiration. To uncouple oxidative phosphorylation, the protonophore carbonyl cyanide p-trifluoromethoxyphenylhydrazone was titrated. The difference between the maximum oxygen consumption flux and the routine respiration represents a reserve range of oxygen flux which is not involved in ATP synthesis or proton leak. In general, the reserve capacity is used upon higher energy demands for ATP^85^ and^35^. Finally, the non-oxidative phosphorylation oxygen flux was determined by blocking the electron transport system with 1 µg/mL antimycin A.

### Electron microscopy

ZIKV-infected brain organoids were immersed in fixative solution containing 2.5% glutaraldehyde (v/v), 0.1 M Na-cacodylate buffer (pH 7.2). All samples were post fixed in 1% OsO4 in cacodylate buffer plus 5 mM calcium chloride and 0.8% potassium ferricyanide, dehydrated in acetone and embedded in EPON. Ultrathin sections (70 nm) were collected on 300 mesh copper grids, stained with uranyl acetate and lead citrate and observed at 80 Kv with a Zeiss 900 transmission electron microscope.

### Nuclear morphometric analysis (NMA)

NMA provides an overview of the main mechanisms of cell proliferation, cell death and mitotic failure^41^. This method was used to assess biological information from nuclear morphometry after ZIKV infection. Images from nuclei were used to assess nuclear heterogeneity and to quantify the intensity of γH2AX and 53BP1 staining. Images were analyzed in Image Pro Plus 6.0, through the acquisition of the following parameters: Area (to represent the nuclear size) and four variables that are used to calculate the Nuclear Irregularity Index, *i.e*. Roundness, Area/Box, Aspect and Radius Ratio. We also acquired two additional variables which are based on mathematical methods to assess the heterogeneity of nuclear staining and the presence of nuclear foci: heterogeneity (determines the fraction of pixel that deviate more than 10% from the average intensity of a given object) and clumpiness (derived from heterogeneity, reflects the object texture, based on the fractions of heterogeneous pixels remaining in an object after an erosion process).

### Comet assay

Alkaline single cell gel (comet assay) was employed to evaluate total DNA damage (single and double strand breaks). Experiments were performed at least in triplicate. The procedure of Singh *et al*.^86^ was followed, with minor modifications. 0.5% Normal-melting agarose (NMA) in PBS was layered onto microscope slides at 64 °C. The slides were stored at RT for at least 24 h. Samples (cell suspension in 120 µL of 1% low melting agarose in PBS) were placed in the slides at 37 °C and immediately covered with a coverslip and left to solidify at 4 °C for 10 min. The coverslip was removed, and the slides were placed on glass cubes and bathed in freshly prepared lysis solution (2.5 M NaCl, 100 mM Na2EDTA, 10 mM Tris with 1% Triton X-100 and 10% DMSO) in the dark for 24 hours at 4 °C. The slides were removed from the lysis solution and placed in a horizontal gel electrophoresis tank filled with fresh alkaline buffer (1 mM Na_2_EDTA and 300 mM NaOH, pH 13) for 20 minutes at 4 °C to allow denaturing and unwinding of the DNA, and the expression of alkaline-labile sites.

Electrophoresis was performed at 25 V and 300 mA for 25 min. to allow the damaged DNA or fragments to migrate towards the anode. Slides were washed three times with Tris HCl 0.4 M for 5 min. and stained with silver solution. Slides were examined at 400x magnification under a light microscope (Leica -ICC50HD). Undamaged cells appeared as nucleoids (class 0) and cells with damaged DNA as comets (classes 1–4). Images of MOCK or infected slides were acquired with Leica LAS EZ software. A double-blind analysis was performed by two experienced observers classifying nucleoids in five damage classes (0–4). About 100 pictures were examined from each slide for the presence of comets, and the damage rate was calculated as the sum of the values obtained times the number of the comet respective class. This sum was then divided by 100, giving the damage rate in arbitrary units.

### ROS analysis

For ROS detection, DHE (ThermoFisher Scientific, #D1168) or mitoSOX^TM^ (ThermoFisher Scientific, #M36008) superoxide indicator dyes were used. MitoTracker^TM^ Green FM (ThermoFisher Scientific, #M7514) and Hoechst 33342 (ThermoFisher Scientific, #H1399) were used to define cytoplasm and nuclei areas, respectively. Images were acquired on Operetta® High-Content Imaging System (Perkin Elmer) and analyzed on Columbus^TM^ Image Data Storage and Analysis System (Perkin Elmer). Cells were incubated with the respective dyes for 30 minutes prior to imaging.

### Statistics

Data comprehend descriptive statistics, and are expressed as mean ± SEM. For two groups comparison, unpaired two-tailed Student’s t- test was performed. For three or more groups, one-way ANOVA with Bonferroni post-test was used. P values are specified at each figure or at their respective legend, and figure symbols represent *p < 0.05, **p < 0.01, ***p < 0.001, ****p < 0.0001.

### Significance statement

Zika virus has caused international concerns due to its association with both microcephaly and Guillain-Barré syndrome. Although it is no longer considered as a global emergency, some basic aspects about Zika virus biology are still unknown. Here we use human iPSC-derived astrocytes, one of the main cell types targeted by Zika virus, to show specific consequences such as reactive gliosis. The infection of astrocytes with Zika virus triggers oxidative stress, mitochondrial dysfunction and DNA damage.

## Supplementary information

Supplementary Material.

## Data Availability

The datasets generated and analyzed during the current study are available from the corresponding author on reasonable request.

## References

[1] Kindhauser MK, Allen T, Frank V, Santhana RS, Dye C (2016). Zika: the origin and spread of a mosquito-borne virus. Bull. World Health Organ..

[2] Dick GWA, Kitchen SF, Haddow AJ (1952). Zika virus. I. Isolations and serological specificity. Trans. R. Soc. Trop. Med. Hyg..

[3] Duffy MR (2009). Zika virus outbreak on Yap Island, Federated States of Micronesia. N. Engl. J. Med..

[4] Jouannic J-M, Friszer S, Leparc-Goffart I, Garel C, Eyrolle-Guignot D (2016). Zika virus infection in French Polynesia. Lancet.

[5] Faria NR (2016). Zika virus in the Americas: Early epidemiological and genetic findings. Sci..

[6] Wang L (2016). From Mosquitos to Humans: Genetic Evolution of Zika Virus. Cell Host Microbe.

[7] Metsky HC (2017). Zika virus evolution and spread in the Americas. Nat..

[8] Cauchemez S (2016). Association between Zika virus and microcephaly in French Polynesia, 2013–15: a retrospective study. Lancet.

[9] Cao-Lormeau VM (2016). Guillain-Barré Syndrome outbreak associated with Zika virus infection in French Polynesia: a case-control study. Lancet.

[10] Qian X (2016). Brain-Region-Specific Organoids Using Mini- bioreactors for Modeling ZIKV Exposure. Cell.

[11] Garcez PP (2016). Zika virus impairs growth in human neurospheres and brain organoids. Sci..

[12] Ming G-L, Tang H, Song H (2016). Advances in Zika Virus Research: Stem Cell Models, Challenges, and Opportunities. Stem Cell.

[13] Dang J (2016). Zika Virus Depletes Neural Progenitors in Human Cerebral Organoids through Activation of the Innate Immune Receptor TLR3. Stem Cell.

[14] Chimelli L (2017). The spectrum of neuropathological changes associated with congenital Zika virus infection. Acta Neuropathol..

[15] Reemst K, Noctor SC, Lucassen PJ, Hol EM (2016). The Indispensable Roles of Microglia and Astrocytes during Brain Development. Front. Hum. Neurosci..

[16] Mlakar J (2016). Zika Virus Associated with Microcephaly. N. Engl. J. Med..

[17] Retallack H (2016). Zika virus cell tropism in the developing human brain and inhibition by azithromycin. Proc. Natl Acad. Sci. USA.

[18] Simonin Y (2016). Zika Virus Strains Potentially Display Different Infectious Profiles in Human Neural Cells. EBioMedicine.

[19] Stefanik M (2018). Characterisation of Zika virus infection in primary human astrocytes. BMC Neurosci..

[20] Jorgačevski J (2019). ZIKV Strains Differentially Affect Survival of Human Fetal Astrocytes versus Neurons and Traffic of ZIKV-Laden Endocytotic Compartments. Sci. Rep..

[21] Potokar M, Jorgačevski J, Zorec R (2019). Astrocytes in Flavivirus Infections. IJMS.

[22] Hamel R (2017). African and Asian Zika virus strains differentially induce early antiviral responses in primary human astrocytes. Infection, Genet. Evolution.

[23] Reid C, Airo A, Hobman T (2015). The Virus-Host Interplay: Biogenesis of +RNA Replication Complexes. Viruses.

[24] Offerdahl DK, Dorward DW, Hansen BT, Bloom ME (2017). Cytoarchitecture of Zika virus infection in human neuroblastoma and Aedes albopictus cell lines. Virology.

[25] Gladwyn-Ng I (2018). Stress-induced unfolded protein response contributes to Zika virus-associated microcephaly. Nat. Neurosci..

[26] Medvedev R, Ploen D, Hildt E (2016). HCV and Oxidative Stress: Implications for HCV Life Cycle and HCV-Associated Pathogenesis. Oxid. Med. Cell Longev..

[27] Fernandez-Garcia M-D, Mazzon M, Jacobs M, Amara A (2009). Pathogenesis of Flavivirus Infections: Using and Abusing the Host Cell. Cell Host Microbe.

[28] Li G (2017). Characterization of cytopathic factors through genome-wide analysis of the Zika viral proteins in fission yeast. Proc. Natl. Acad. Sci. USA.

[29] Nem de Oliveira Souza I (2018). Acute and chronic neurological consequences of early-life Zika virus infection in mice. Sci. Transl. Med..

[30] Souza BSF (2016). Zika virus infection induces mitosis abnormalities and apoptotic cell death of human neural progenitor cells. Sci. Rep..

[31] Onorati M (2016). Zika Virus Disrupts Phospho-TBK1 Localization and Mitosis in Human Neuroepithelial Stem Cells and Radial Glia. CellReports.

[32] Vitale I, Galluzzi L, Castedo M, Kroemer G (2011). Mitotic catastrophe: a mechanism for avoiding genomic instability. Nat. Rev. Mol. Cell Biol..

[33] Ryan E, Hollingworth R, Grand R (2016). Activation of the DNA Damage Response by RNA Viruses. Biomolecules.

[34] Devhare P, Meyer K, Steele R, Ray RB, Ray R (2017). Zika virus infection dysregulates human neural stem cell growth and inhibits differentiation into neuroprogenitor cells. Cell Death Dis..

[35] Garcez PP (2017). Zika virus disrupts molecular fingerprinting of human neurospheres. Sci. Rep..

[36] Wu J, Lu L-Y, Yu X (2010). The role of BRCA1 in DNA damage response. Protein Cell.

[37] Kuo LJ, Yang L-X (2008). Gamma-H2AX - a novel biomarker for DNA double-strand breaks. Vivo.

[38] Casas, B. S. *et al*. hiPSC-derived neural stem cells from patients with schizophrenia induce an impaired angiogenesis. *Translational Psychiatry* 1–15, 10.1038/s41398-018-0095-9 (2018). 10.1038/s41398-018-0095-9 PMC5821759 29467462

[39] Yan Y (2013). Efficient and Rapid Derivation of Primitive Neural Stem Cells and Generation of Brain Subtype Neurons From Human Pluripotent Stem Cells. STEM CELLS Transl. Med..

[40] Donald CL (2016). Full Genome Sequence and sfRNA Interferon Antagonist Activity of Zika Virus from Recife, Brazil. PLoS Negl. Trop. Dis..

[41] Filippi-Chiela EC (2012). Nuclear morphometric analysis (NMA): screening of senescence, apoptosis and nuclear irregularities. PLoS ONE.

[42] Cortese M (2017). Ultrastructural Characterization of Zika Virus Replication Factories. Cell Reports.

[43] Fleury C, Mignotte B, Vayssière J-L (2002). Mitochondrial reactive oxygen species in cell death signaling. Biochim..

[44] Ouyang Y-B, Xu L-J, Emery JF, Lee AS, Giffard RG (2011). Overexpressing GRP78 influences Ca2+ handling and function of mitochondria in astrocytes after ischemia-like stress. MITOCH.

[45] Lieber MR (2010). The Mechanism of Double-Strand DNA Break Repair by the Nonhomologous DNA End-Joining Pathway. Annu. Rev. Biochem..

[46] Caldecott KW (2008). Single-strand break repair and genetic disease. Nat. Rev. Genet..

[47] Schneider L, Fumagalli M, di Fagagna FDAA (2011). Terminally differentiated astrocytes lack DNA damage response signaling and are radioresistant but retain DNA repair proficiency. Cell Death Differ..

[48] Panier S, Boulton SJ (2014). Double-strand break repair: 53BP1 comes into focus. Nat. Rev. Mol. Cell Biol..

[49] Nakamura AJ, Rao VA, Pommier Y, Bonner WM (2014). The complexity of phosphorylated H2AX foci formation and DNA repair assembly at DNA double-strand breaks. Cell Cycle.

[50] Zhang X-P, Liu F, Cheng Z, Wang W (2009). Cell fate decision mediated by p53 pulses. Proc. Natl. Acad. Sci. USA.

[51] Liddelow SA, Barres BA (2017). Reactive Astrocytes: Production, Function, and Therapeutic Potential. Immun..

[52] Bender, C., Frik, J. & Gómez, R. M. In *Astrocytes* 109–124 (Nova Biomedical), 10.13140/2.1.4745.8243 (2012).

[53] Trindade, P. *et al*. Short and long TNF-alpha exposure recapitulates canonical astrogliosis events in human induced pluripotent stem cells-derived astrocytes. *bioRxiv* 1–37, 10.1101/722017 (2019). 10.1002/glia.23786 32003513

[54] Meertens L (2017). Axl Mediates ZIKA Virus Entry in Human Glial Cells and Modulates Innate Immune Responses. Cell Reports.

[55] Richard AS (2017). AXL-dependent infection of human fetal endothelial cells distinguishes Zika virus from other pathogenic flaviviruses. Proc. Natl. Acad. Sci. USA.

[56] Liu S, DeLalio LJ, Isakson BE, Wang TT (2016). AXL-Mediated Productive Infection of Human Endothelial Cells by Zika Virus Novelty and Significance. Circ. Res..

[57] Wang J (2018). Zika virus infected primary microglia impairs NPCs proliferation and differentiation. Biochem. Biophys. Res. Commun..

[58] Smith DR (2017). Neuropathogenesis of Zika Virus in a Highly Susceptible Immunocompetent Mouse Model after Antibody Blockade of Type I Interferon. PLoS Negl. Trop. Dis..

[59] Sher AA, Glover KKM, Coombs KM (2019). Zika Virus Infection Disrupts Astrocytic Proteins Involved in Synapse Control and Axon Guidance. Front. Microbiol..

[60] Molofsky AV (2012). Astrocytes and disease: a neurodevelopmental perspective. Genes. Dev..

[61] Phatnani, H. & Maniatis, T. Astrocytes in neurodegenerative disease. *Cold Spring Harb Perspect Biol* **7** (2015). 10.1101/cshperspect.a020628 PMC4448607 25877220

[62] Seifert G, Schilling K, Steinhäuser C (2006). Astrocyte dysfunction in neurological disorders: a molecular perspective. Nat. Rev. Neurosci..

[63] Yu CY, Hsu YW, Liao CL, Lin YL (2006). Flavivirus Infection Activates the XBP1 Pathway of the Unfolded Protein Response To Cope with Endoplasmic Reticulum Stress. J. Virol..

[64] Gullberg RC, Jordan Steel J, Moon SL, Soltani E, Geiss BJ (2015). Oxidative stress influences positive strand RNA virus genome synthesis and capping. Virology.

[65] Jheng J-R, Ho J-Y, Horng J-T (2014). ER stress, autophagy, and RNA viruses. Front. Microbiol..

[66] Blázquez A-B, Escribano-Romero E, Merino-Ramos T, Saiz J-C, Martín-Acebes MA (2014). Stress responses in flavivirus-infected cells: activation of unfolded protein response and autophagy. Front. Microbiol..

[67] Desler C (2012). Is There a Link between Mitochondrial Reserve Respiratory Capacity and Aging?. J. Aging Res..

[68] Madigan CA (2017). A Macrophage Response to Mycobacterium leprae Phenolic Glycolipid Initiates Nerve Damage in Leprosy. Cell.

[69] Chandler AL (2012). Neural tube defects and maternal intake of micronutrients related to one-carbon metabolism or antioxidant activity. Birth Defects Res. Part. A Clin. Mol. Teratol..

[70] O’Driscoll M, Jeggo PA (2008). The role of the DNA damage response pathways in brain development and microcephaly: Insight from human disorders. DNA Repair..

[71] Mao Z, Bozzella M, Seluanov A, Gorbunova V (2008). Comparison of nonhomologous end joining and homologous recombination in human cells. DNA Repair..

[72] van Gent DC, Hoeijmakers JH, Kanaar R (2001). Chromosomal stability and the DNA double-stranded break connection. Nat. Rev. Genet..

[73] Morgan WF (1998). DNA double-strand breaks, chromosomal rearrangements, and genomic instability. Mutat. Res..

[74] Lambrus BG (2016). A USP28–53BP1–p53–p21 signaling axis arrests growth after centrosome loss or prolonged mitosis. J. Cell Biol..

[75] Nigg EA, Holland AJ (2018). Once and only once: mechanisms of centriole duplication and their deregulation in disease. Nat. Rev. Mol. Cell Biol..

[76] Vitale I (2010). Illicit survival of cancer cells during polyploidization and depolyploidization. Cell Death Differ..

[77] Zody MC (2006). DNA sequence of human chromosome 17 and analysis of rearrangement in the human lineage. Nat..

[78] Mladinich, M. C., Schwedes, J. & Mackow, E. R. Zika Virus Persistently Infects and Is Basolaterally Released from Primary Human Brain Microvascular Endothelial Cells. *mBio* **8**, e00952–17–17 (2017). 10.1128/mBio.00952-17 PMC5513708 28698279

[79] Mulkey SB, Arroyave-Wessel M, Peyton C, *et al*. Neurodevelopmental Abnormalities in Children With In Utero Zika Virus Exposure Without Congenital Zika Syndrome [published online ahead of print, 2020 Jan 6]. *JAMA Pediatr*. 10.1001/jamapediatrics.2019.5204 (2020). 10.1001/jamapediatrics.2019.5204 PMC6990858 31904798

[80] Schreiner B (2015). Astrocyte Depletion Impairs Redox Homeostasis and Triggers Neuronal Loss in the Adult CNS. CellReports.

[81] Guo C, Sun L, Chen X, Zhang D (2013). Oxidative stress, mitochondrial damage and neurodegenerative diseases. Neural Regen. Res..

[82] Bhat AH (2015). Oxidative stress, mitochondrial dysfunction and neurodegenerative diseases; a mechanistic insight. Biomedicine et. Pharmacotherapy.

[83] Sochacki J (2016). Generation of iPS cell lines from schizophrenia patients using a non-integrative method. Stem Cell Res..

[84] Sochacki J, Devalle S, Reis M, Fontenelle LF, Rehen S (2016). Generation of urine iPS cell line from a patient with obsessive-compulsive disorder using a non-integrative method. Stem Cell Res..

[85] Gnaiger, E. *Mitochondrial Pathways and Respiratory Control* (2012).

[86] Singh NP, McCoy MT, Tice RR, Schneider EL (1988). A simple technique for quantitation of low levels of DNA damage in individual cells. Exp. Cell Res..

