# Supplementary material for: Zika virus infection leads to mitochondrial failure, oxidative stress and DNA damage in human iPSC-derived astrocytes
Source: Sci Rep. 2020 Jan 27;10:1218. doi: 10.1038/s41598-020-57914-x (PMC6985105; doi:10.1038/s41598-020-57914-x)
Supplement: Supplementary file 1 — Supplementary Material. [file 41598_2020_57914_MOESM1_ESM.pdf]

Supplementary Material

Zika virus infection leads to mitochondrial failure, oxidative stress and DNA damage in human iPSC-derived astrocytes

Pítia Flores Ledur [1†], Karina Karmirian [1,2†], Carolina da Silva Gouveia Pedrosa [1], Leticia Rocha Quintino Souza [1], Gabriela Assis de Lemos [3], Thiago Martino Martins [2], Jéssica de Cassia Cavalheiro Gomes Ferreira [2], Gabriel Ferreira de Azevedo Reis [4], Eduardo Santos Silva [4], Débora Silva [5], José Alexandre Salerno[1,2], Isis Moraes Ornelas[1], Sylvie Devalle [1], Rodrigo Furtado Madeiro da Costa [1] , Livia Goto-Silva [1], Luiza Mendonça Higa [6], Adriana Melo [8], Amilcar Tanuri [6], Leila Chimelli [5], Marcos Massao Murata [4], Patrícia Pestana Garcez [2], Eduardo Cremonese Filippi-Chiela [7], Antonio Galina [3], Helena Lobo Borges [2], Stevens Kastrup Rehen [1,2]*

1. D'Or Institute for Research and Education, Rio de Janeiro, Brazil
2. Institute of Biomedical Sciences, Federal University of Rio de Janeiro (UFRJ), Rio de Janeiro, RJ, Brazil
3. Institute of Medical Biochemistry Leopoldo De Meis, Federal University of Rio de Janeiro, Rio de Janeiro, RJ, Brazil
4. Insitute of Biology, Department of Biophysics and Biometrics, State University of Rio de Janeiro (UERJ), Rio de Janeiro, RJ, Brazil
5. Laboratory of Neuropathology, State Institute of Brain Paulo Niemeyer and Federal University of Rio de Janeiro (UFRJ), Rio de Janeiro, RJ, Brazil
6. Institute of Biology, Federal University of Rio de Janeiro (UFRJ), Rio de Janeiro, RJ, Brazil
7. Institute of Health Sciences, Federal University of Rio Grande do Sul (UFRGS), Porto Alegre, RS, Brazil
8. Research Institute Prof. Joaquim Amorim Neto (IPESQ), Campina Grande, PB, Brazil

† The two authors contributed equally to this work.

*Corresponding author - D'Or Institute for Research and Education – Rua Diniz Cordeiro, 30. Botafogo, Rio de Janeiro – RJ – CEP 22281-100. srehen@lance-ufrj.org

Legends to the Figures:

1. Characterization of human iPSC-derived astrocytes. **A.** Immunocytochemistry staining to main astrocytic markers (ALDH1L1, EAAT1, EAAT2, GFAP, S100β, and Vimentin). **B.** Western blotting analysis of GFAP in iPSC-derived astrocyte and positive control (astroglioma cell line U87MG - ATCC® HTB14™).

2. Nuclear Morphometric Analysis of ZIKV-infected cells. **A**. NMA results from MOCK and ZIKV-infected NSC, Astrocytes and Neurons. Each nucleus is classified based on morphometry as Normal (N), Small and Regular (SR) or Irregular (I). **B**. Nuclear morphometric analysis of DHE stained MOCK and ZIKV-infected astrocytes. Red diamond-shaped markers represent cells with high (positive) DHE nuclei staining; white diamond markers represent low (negative) DHE staining. **C**. DHE levels in ZIKV-infected astrocytes. The intensity of DHE staining to each group (MOCK, ZIKV and ZIKV+AA) is shown on the left. Based on the average of MOCK group, we determined the percentage of cells with high levels of DHE (*i.e.* cells with DHE levels higher than the average) and low levels of DHE (*i.e.* cells with DHE levels lower than the average). We also determined the percentage of cells showing very high levels of DHE (*i.e.* cells with DHE levels higher than the average for MOCK + 1 standard deviation) and very low levels of DHE (*i.e.* cells with DHE levels lower than the average for MOCK – 1 standard deviation). **D**. Nuclear morphometric analysis shows the percentage of regular, irregular and small regular nuclei of MOCK and ZIKV-infected astrocytes treated with ascorbic acid.

3. ZIKV infection in mice. Brain slices from ZIKV and MOCK infected mice were evaluated. Microglial cells (IBA1+ cells in **A**), astrocytes (ALDH1L1+ cells in **B**), neurons (NeuN+ cells in **C**) and cells from the oligodendroglial lineage (OLIG2+ cells in **D**) were analyzed in the cingulate cortex. Neural stem cells (SOX2+) were evaluated in the hippocampus in **E** and shown in detail in **F**. Percent of infection is shown in **G** and represents the percentage of % NS1 positive cells among each cell population. At least 6 animals were analyzed for the counts of the cingulate cortex and 3 animals for hippocampus.

4. Electron Microscopy (EM) images of ZIKV-infected brain organoids. **A**. EM images of mitochondrion in MOCK and ZIKV-infected astrocytes. **B.** Section of a brain organoid infected by ZIKV, where NS1 staining overlaps with S100b staining. **D.** Shows the entire organoid.

5. ROS production in ZIKV infection. Representative images of mitoSOX ROS superoxide indicator dye staining in MOCK and ZIKV-infected astrocytes untreated or treated with ascorbic acid. Nuclei are stained with HOECHST. N = 3.

6. Vimentin intensity increase in ZIKV-induced gliosis. **A**. Representative images of vimentin and NS1 staining of astrocytes in MOCK, ZIKV MOI 0.125 and TNF-α conditions. Vimentin intensity was quantified in **B**. N = 4.

7. Full-length Western Blots Membranes. **A.** Detection of 53BP1, γH2Ax and Actin proteins in MOCK- and ZIKV-infected astrocytes. A precasted gel (4-20%) was used. The last 4 lanes (from left to right) are MOCK 24hpi, ZIKV 24hpi, MOCK 48hpi and ZIKV 48hpi, respectively. Three different proteins (with distinct molecular weights) were detected within the same gel/membrane (therefore membranes were cut). After dying the membrane (using Ponceau dye), membrane was cut in three regions for specific antibody

incubation, according to the molecular weight indicator in the ladder (Kaleidoscope cat#161-0375 Bio-Rad) to avoid stripping process. It is important to mention that all three pieces of membranes are complementary, and when putting the membranes altogether the edges fit as a puzzle. **B.** Membranes used for detection of GFAP and Actin. Full-range rainbow marker (Amershan, #RPN800E) was ran in the first lane to allow identification of the respective molecular weight intervals. The same process described above were performed after proteins were transferred to membrane to avoid stripping and reprobing.

8. Characterization of iPSCs pluripotency. **A.** Colonies immunostained for self-renewal markers Nanog, Sox-2, Oct-3/4, TRA-1-60, TRA-1-81 and SSEA-4. **B.** Immunostaining of cells dissociated from embryoid-bodies for germ layer markers AFP (endoderm) and SMA (mesoderm). **C.** b-TUB-III (ectoderm) immunostaining of migrating neural cells.

Figure S1

A.

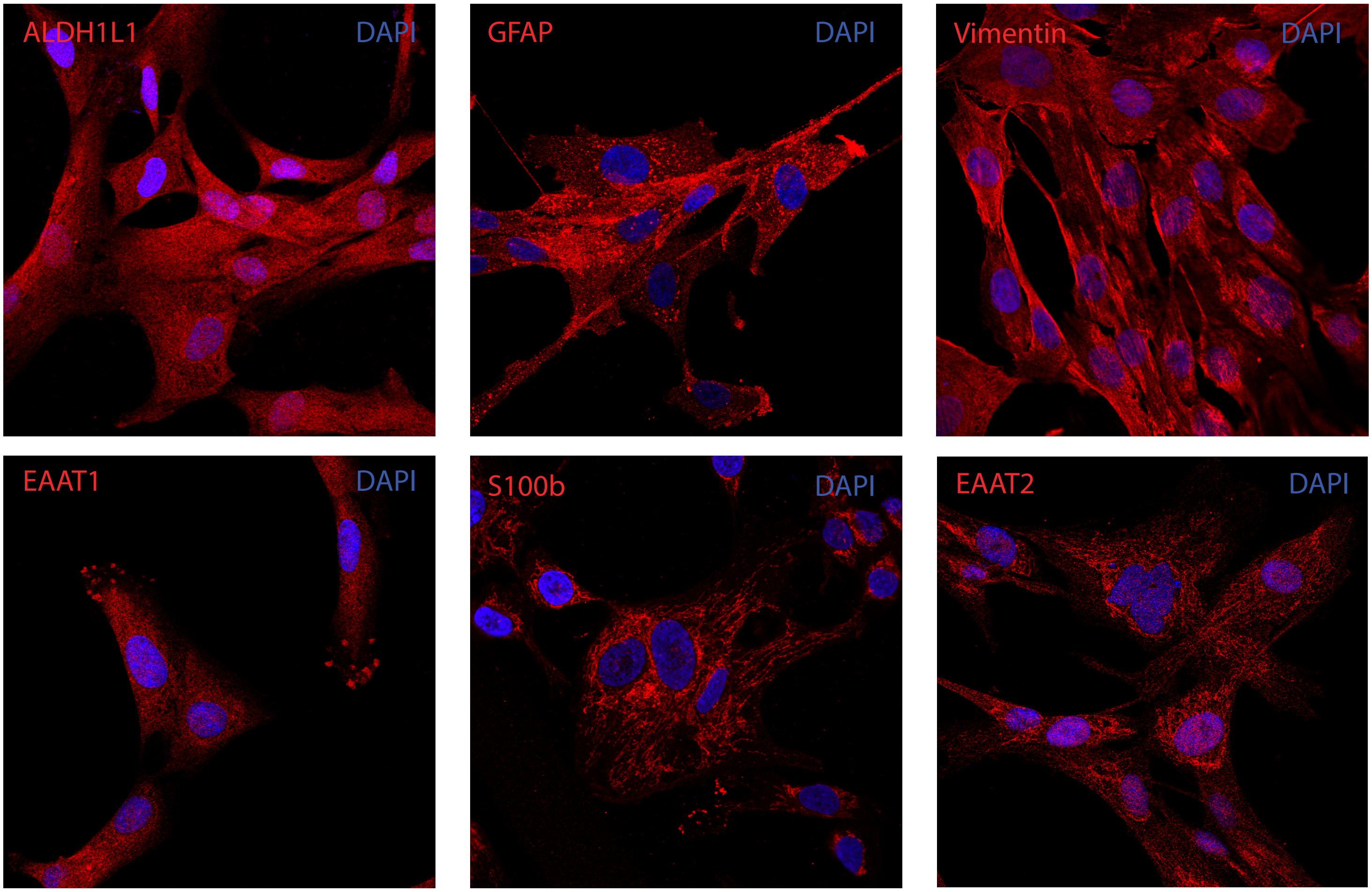
ALDH1L1
DAPI
GFAP
DAPI
Vimentin
DAPI
EAAT1
DAPI
S100b
DAPI
EAAT2
DAPI

B.

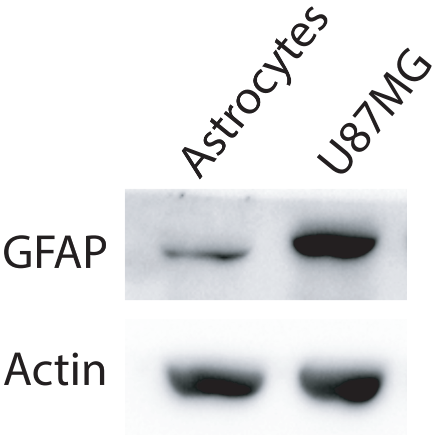
Astrocytes
U87MG
GFAP
Actin

Figure S2

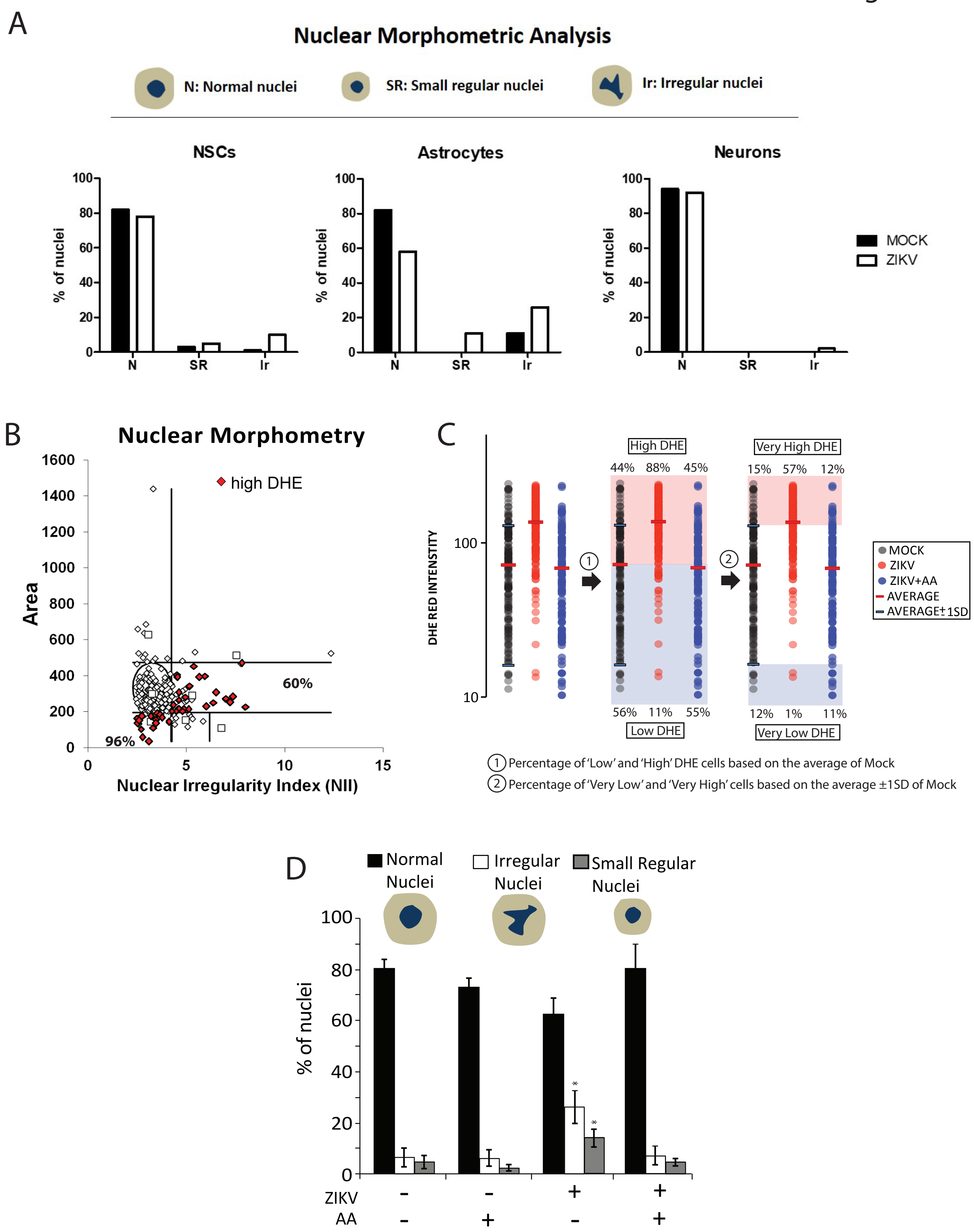

A
Nuclear Morphometric Analysis
N: Normal nuclei
SR: Small regular nuclei
Ir: Irregular nuclei
NSCs
Astrocytes
Neurons
% of nuclei
100
80
60
40
20
0
N
SR
Ir
MOCK
ZIKV
B
Nuclear Morphometry
high DHE
Area
1600
1400
1200
1000
800
600
400
200
0
60%
96%
0
5
10
15
Nuclear Irregularity Index (NII)
C
DHE RED INTENSITY
100
10
High DHE
44% 88% 45%
56% 11% 55%
Low DHE
Very High DHE
15% 57% 12%
12% 1% 11%
Very Low DHE
MOCK
ZIKV
ZIKV+AA
AVERAGE
AVERAGE±1SD
1 Percentage of 'Low' and 'High' DHE cells based on the average of Mock
2 Percentage of 'Very Low' and 'Very High' cells based on the average ±1SD of Mock
D
Normal Nuclei
Irregular Nuclei
Small Regular Nuclei
% of nuclei
100
80
60
40
20
0
ZIKV - - + +
AA - + - +

Figure S3

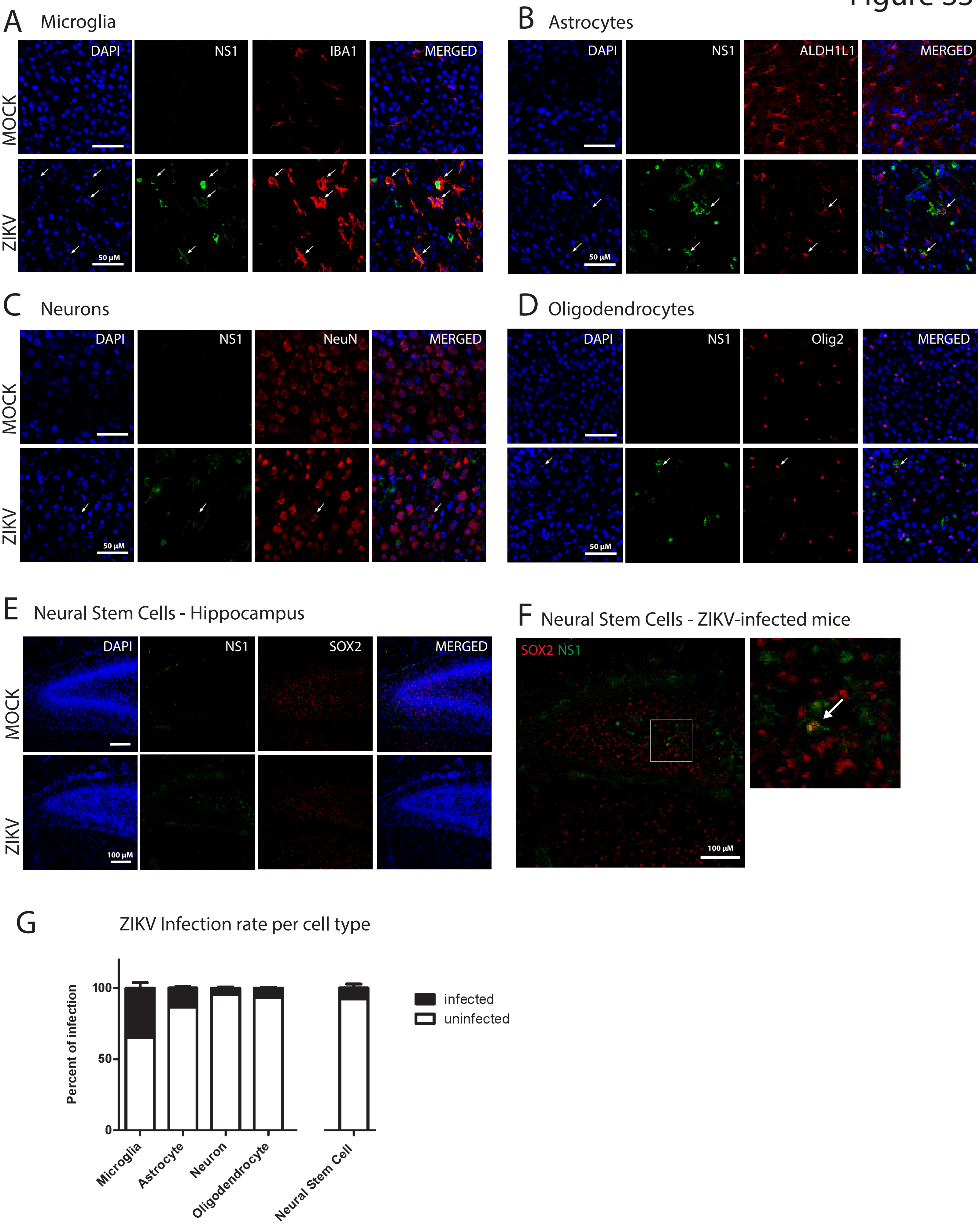
A Microglia
DAPI
NS1
IBA1
MERGED
MOCK
ZIKV
50 µM
B Astrocytes
DAPI
NS1
ALDH1L1
MERGED
MOCK
ZIKV
50 µM
C Neurons
DAPI
NS1
NeuN
MERGED
MOCK
ZIKV
50 µM
D Oligodendrocytes
DAPI
NS1
Olig2
MERGED
MOCK
ZIKV
50 µM
E Neural Stem Cells - Hippocampus
DAPI
NS1
SOX2
MERGED
MOCK
ZIKV
100 µM
F Neural Stem Cells - ZIKV-infected mice
SOX2 NS1
100 µM
G ZIKV Infection rate per cell type
Percent of infection
100
50
0
Microglia
Astrocyte
Neuron
Oligodendrocyte
Neural Stem Cell
infected
uninfected

Figure S4

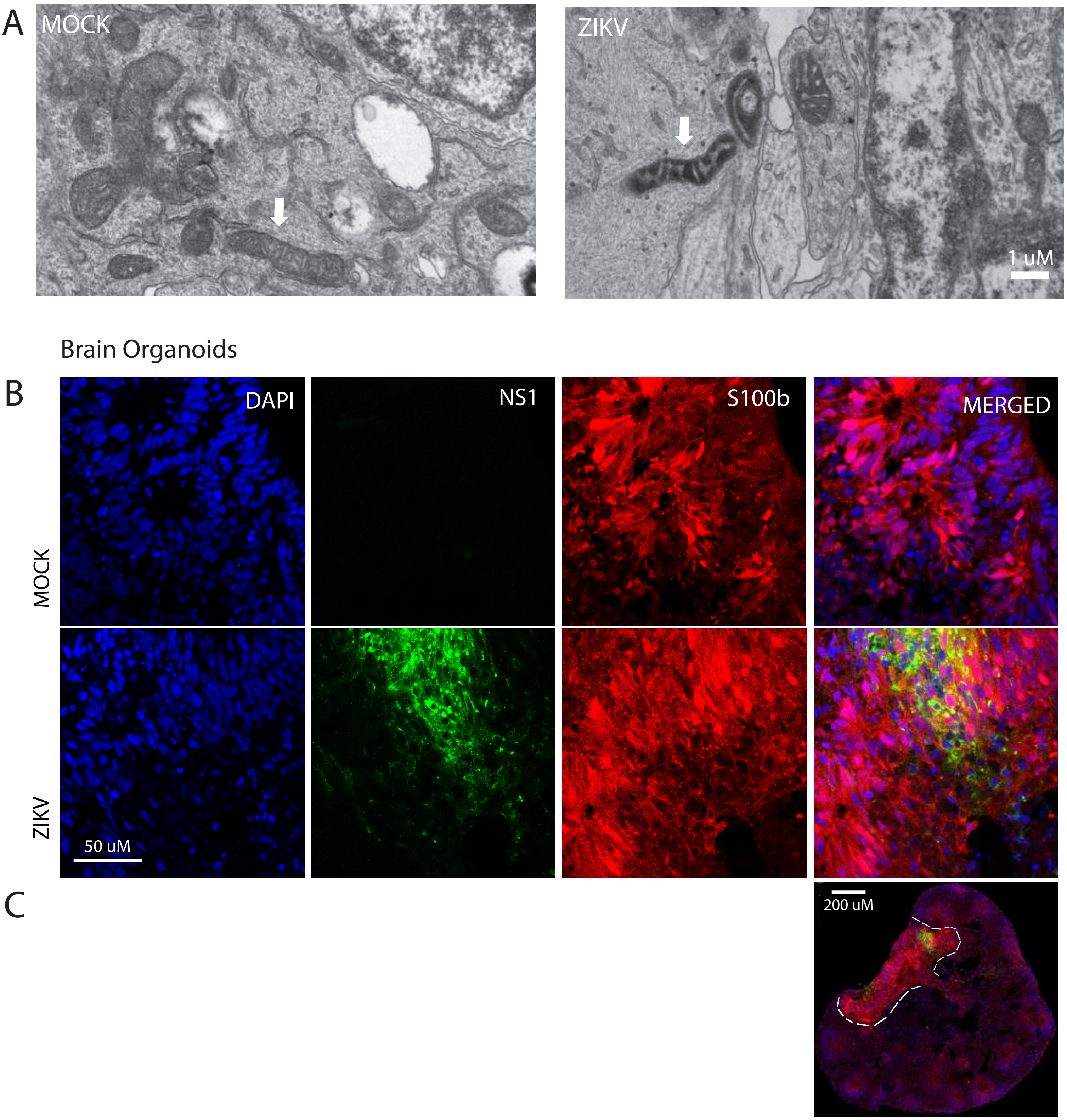
A
MOCK
ZIKV
1 uM
Brain Organoids
B
DAPI
NS1
S100b
MERGED
MOCK
ZIKV
50 uM
C
200 uM

Figure S5

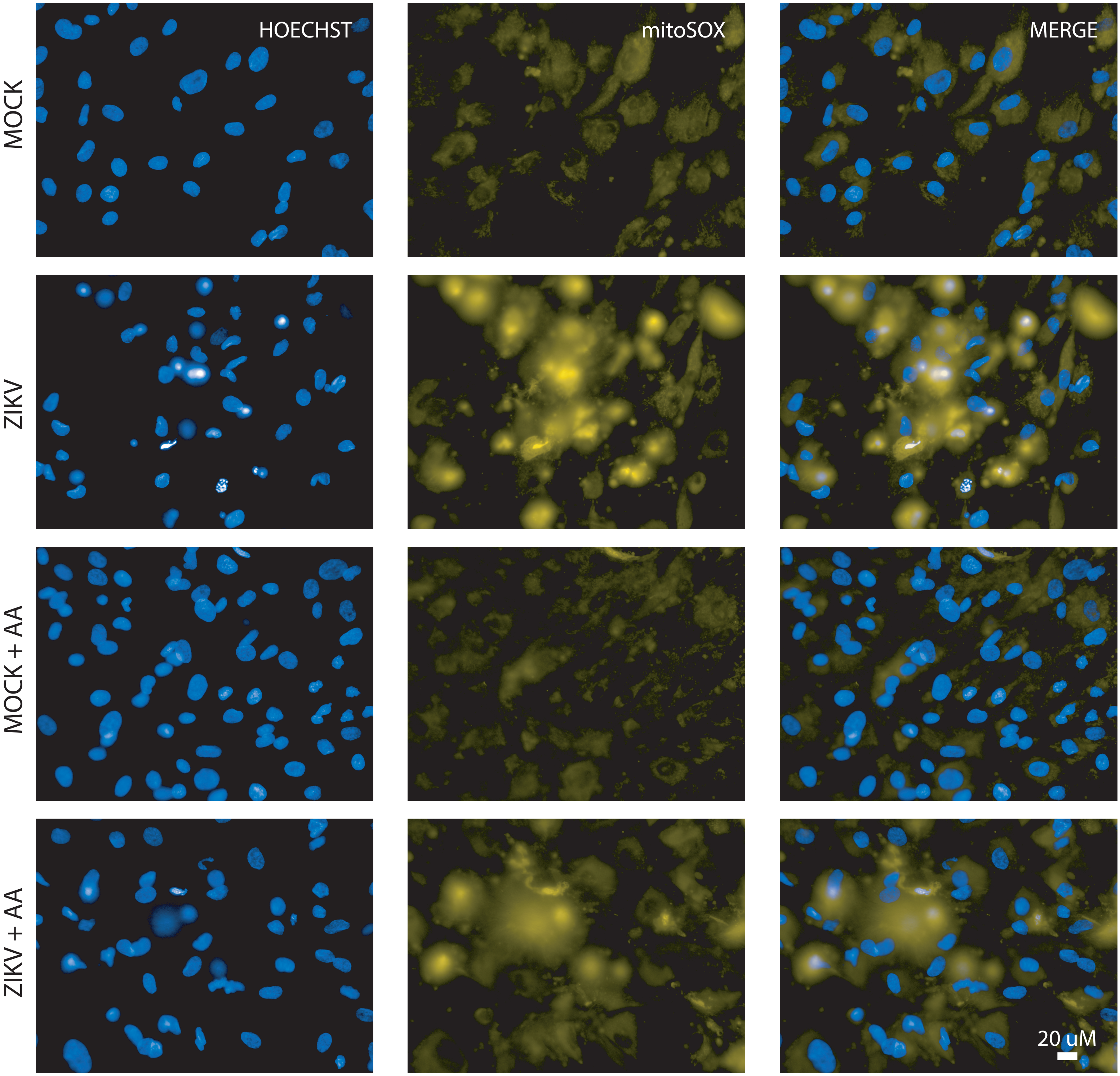

Figure S6

A.

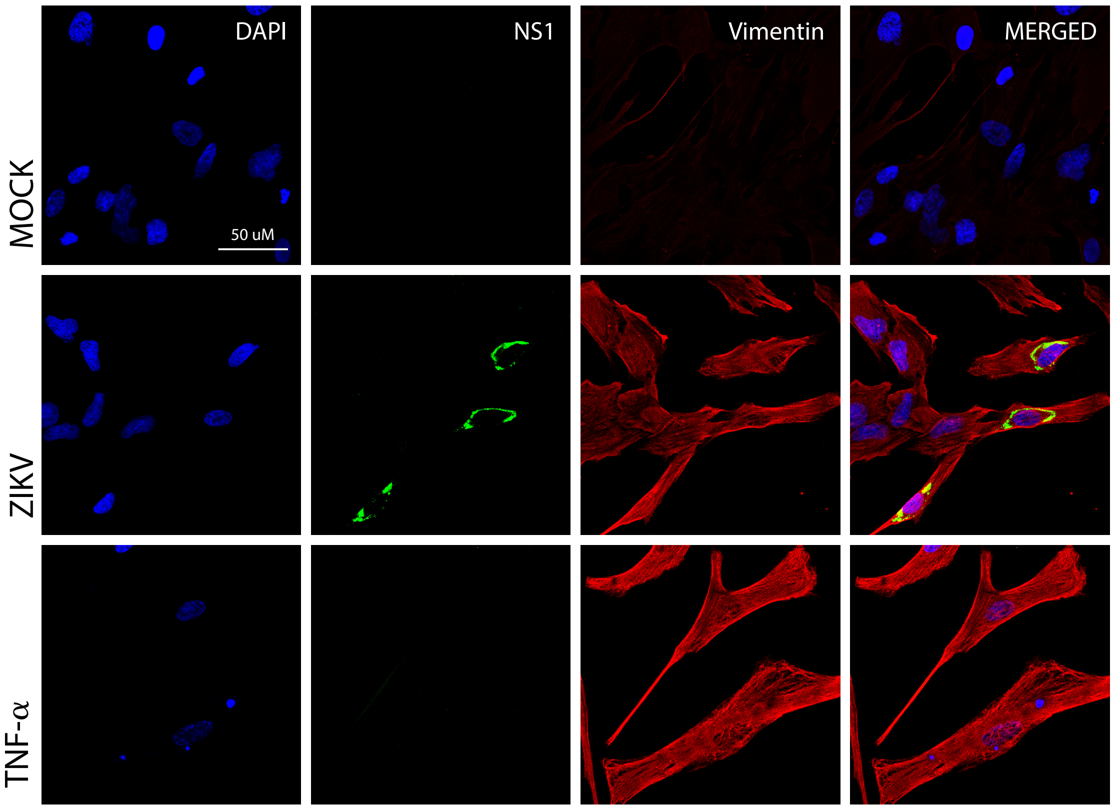
DAPI
NS1
Vimentin
MERGED
MOCK
ZIKV
TNF-α
50 uM

B.

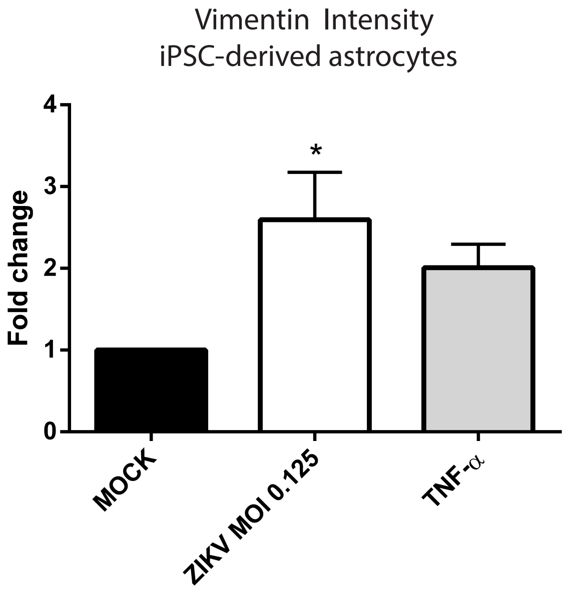
Vimentin Intensity
iPSC-derived astrocytes
Fold change
0
1
2
3
4
*
MOCK
ZIKV MOI 0.125
TNF-α

Figure S7

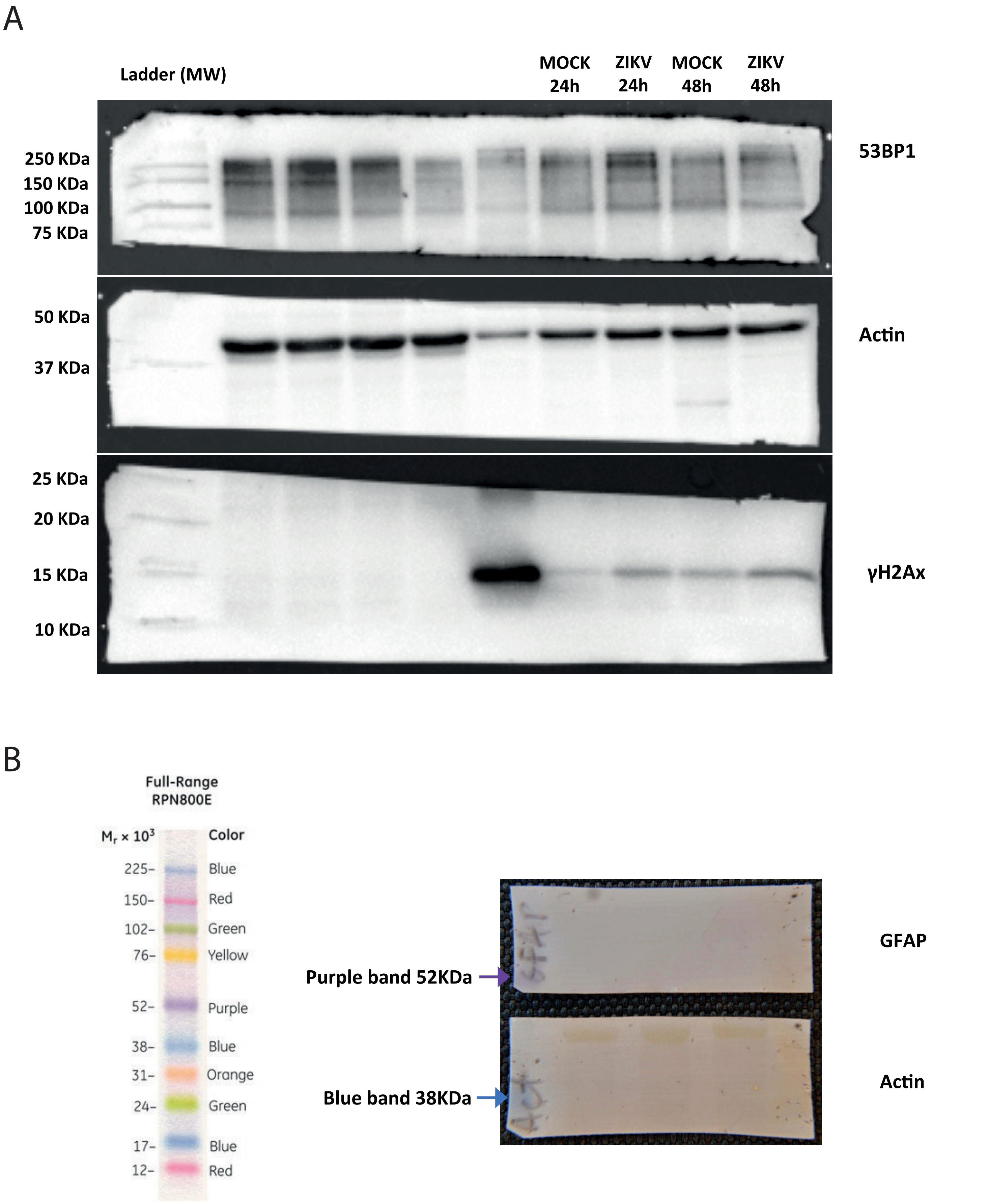

A
Ladder (MW)
MOCK 24h
ZIKV 24h
MOCK 48h
ZIKV 48h
250 KDa
150 KDa
100 KDa
75 KDa
53BP1
50 KDa
37 KDa
Actin
25 KDa
20 KDa
15 KDa
10 KDa
γH2Ax
B
Full-Range
RPN800E
Mr × 10³
Color
225– Blue
150– Red
102– Green
76– Yellow
52– Purple
38– Blue
31– Orange
24– Green
17– Blue
12– Red
Purple band 52KDa
Blue band 38KDa
GFAP
Actin

Figure S8

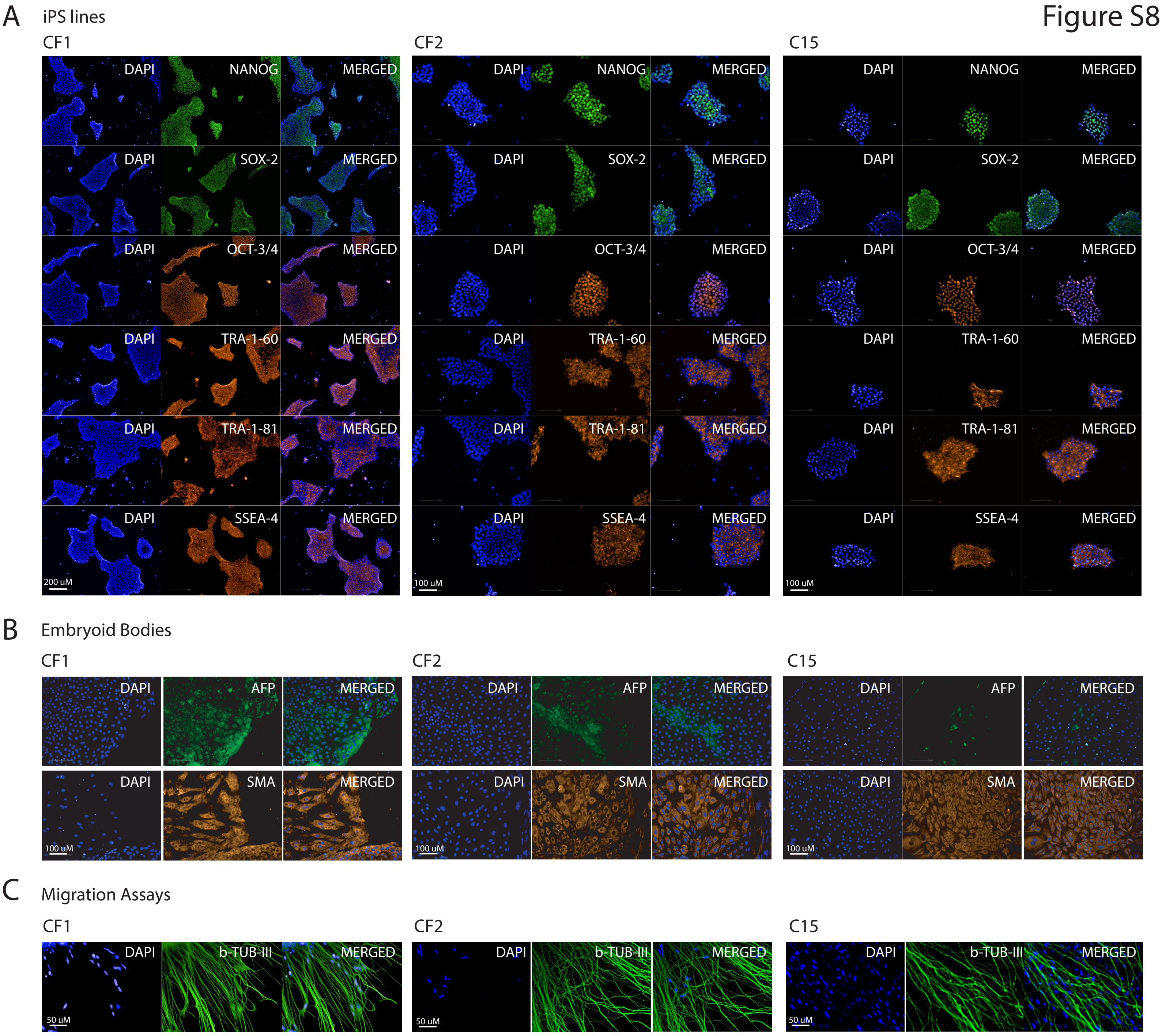
A
iPS lines
CF1
CF2
C15
DAPI
NANOG
MERGED
SOX-2
OCT-3/4
TRA-1-60
TRA-1-81
SSEA-4
200 uM
100 uM
B
Embryoid Bodies
AFP
SMA
C
Migration Assays
b-TUB-III
50 uM
